# Severe multi-year drought coincident with Hittite collapse around 1198–1196 bc

**DOI:** 10.1038/s41586-022-05693-y

**Published:** 2023-02-08

**Authors:** Sturt W. Manning, Cindy Kocik, Brita Lorentzen, Jed P. Sparks

**Affiliations:** 1 Cornell Tree-Ring Laboratory, Department of Classics and Cornell Institute of Archaeology and Material Studies, Cornell University, Ithaca, NY USA; 2 The Cyprus Institute, Nicosia, Cyprus; 3 Mississippi Valley Archaeology Center, University of Wisconsin-La Crosse, La Crosse, WI USA; 4 Department of Anthropology, University of Georgia, Athens, GA USA; 5 Department of Ecology and Evolutionary Biology, Cornell University, Ithaca, NY USA

**Keywords:** Palaeoclimate, Archaeology

## Abstract

The potential of climate change to substantially alter human history is a pressing concern, but the specific effects of different types of climate change remain unknown. This question can be addressed using palaeoclimatic and archaeological data. For instance, a 300-year, low-frequency shift to drier, cooler climate conditions around 1200 bc is frequently associated with the collapse of several ancient civilizations in the Eastern Mediterranean and Near East^1–4^. However, the precise details of synchronized climate and human-history-scale associations are lacking. The archaeological–historical record contains multiple instances of human societies successfully adapting to low-frequency climate change^5–7^. It is likely that consecutive multi-year occurrences of rare, unexpected extreme climatic events may push a population beyond adaptation and centuries-old resilience practices^5,7–10^. Here we examine the collapse of the Hittite Empire around 1200 bc. The Hittites were one of the great powers in the ancient world across five centuries^11–14^, with an empire centred in a semi-arid region in Anatolia with political and socioeconomic interconnections throughout the ancient Near East and Eastern Mediterranean, which for a long time proved resilient despite facing regular and intersecting sociopolitical, economic and environmental challenges. Examination of ring width and stable isotope records obtained from contemporary juniper trees in central Anatolia provides a high-resolution dryness record. This analysis identifies an unusually severe continuous dry period from around 1198 to 1196 (±3) bc, potentially indicating a tipping point, and signals the type of episode that can overwhelm contemporary risk-buffering practices.

## Abstract (web summary)

Tree-ring analysis is correlated with stable isotope data and historical texts to identify an unusual multi-year period of drought from around 1198 to 1196 bc associated with the collapse of the Hittite Empire.

## Main

**Extended Data Fig. 1:**
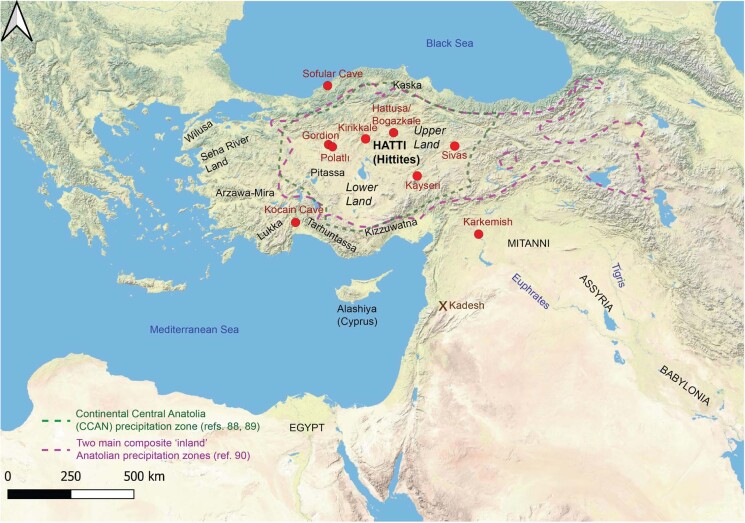
Map of East Mediterranean-Near East indicating the major ancient empires (Hatti = Hittites, Egypt, Mitanni, Assyria, Babylonia), several other main political entities sometimes within, sometimes on the margins of, the Hittite Empire (Kaska, Wilusa, Seha River Land, Arzawa-Mira, Lukka, Pitassa, Tarhuntassa, Alashiya, Kizzuwatna)^11,12^, the main sites/locations mentioned in this paper (Hattusa next to modern Boğazkale, Gordion, Polatlı, Sofular Cave, Kocain Cave, Kirikkale, Sivas, Kayseri, Karkemish, Kadesh), and the approximate Continental Central Anatolia (CCAN) precipitation zone^88,89^ or the combined two main simplified composite ‘inland’ precipitation regimes identified for Anatolia in ref. ^90^. Note: Wiyanawanda, Masa, and Ikkuna, mentioned in the main text, are all thought to lie in, or close to, the Lukka territory in southwest Anatolia^11^. Map created from free and open (public domain) Natural Earth data (https://www.naturalearthdata.com/), using the Natural Earth II dataset.

The vast Hittite Kingdom and subsequently Empire, based in central Anatolia, Turkey, with its capital at Hattusa, is recognized from both rich archaeological remains and textual sources as one of the major Old World powers of the Eastern Mediterranean and Near East between 1650 and 1200 bc. At its apex, the Hittite Empire maintained control over central, southern and southeastern Anatolia, the northern Levant and northern Syria, with almost all of Anatolia being under the Hittite sphere of influence (Extended Data Fig. 1). During this time, the Hittite Empire vied with the Egyptian Empire for sociopolitical dominance in the Near East, a struggle that culminated in the largest battle of the era at Kadesh in Syria in the early 13th century bc^15^.

Around or shortly after 1200 bc, the Hittite Empire and central administrative system collapsed in a great realignment that reverberated around the Near East^4,11–13,16–20^. The reign of the last known king, Suppiluliuma II, began around 1207 bc and included claimed victories against several intra-Anatolian rivals (Wiyanawanda, Masa, Lukka and Ikkuna) and Alashiya (Cyprus) in sea and land battles, but no further Hittite rulers were recorded subsequently. An inscription of the Egyptian ruler Ramesses III—approximately dated to 1188 bc or 1177 bc, depending on selection and debate in Egyptian history and chronology—lists the Hittites among those swept away by the ‘Sea Peoples’ before they attacked Egypt^4,11,17,18^.

The end of settlement at Hattusa itself has been a key topic of historical scrutiny. Long considered a victim of attack, whether by the Sea Peoples or local Anatolian raiders, archaeological investigations now indicate that the city was abandoned and emptied by the royal administration and only later burnt^11,12,16,21–23^. Hattusa was the centripetal political and core religious venue of the Hittite gods and kings for centuries, and the reasons for its abandonment remain unclear. The Hittite central sociopolitical and economic system withstood multiple, diverse crises during its tenure: intra-Anatolian tensions with Kaska invaders, rivalries and tensions among constituent elements of the Empire (Arzawa-Mira, Tarhuntassa and Karkemish), dynastic politics (rivals and usurpers to the Great King), and threats such as plague, as well as external challenges, are regularly evident in the Empire’s history^11–16,21–25^. However, the final collapse and abandonment of Hattusa and the central Hittite administration (and thus the cessation of written historical documents) was different and seismic in its scale of impact. Despite evidence of adaptation and continuation of elements of Hittite political and cultural systems at various loci within former Hittite territories (especially in the southeast and in northern Syria)^26–28^, it is undeniable that the succeeding neo-Hittite states that emerged were of a very different, much smaller scale and character^19,24–29^. As Middleton recently stated regarding Hattusa’s collapse: “A context of conflict was nothing new … and so it seems appropriate to conclude that something historically specific (and perhaps never recoverable) happened to precipitate the abandonment”^22^.

Recent scholarship inspired by contemporary concerns around climate change has increasingly shifted from explaining the fall of the Hittites and the wider collapse of several Late Bronze Age civilizations with invaders or raiders, earthquakes or various political–economic changes as prime movers, to speculate instead on a possible underlying climatic or environmental driver^1–4,11,30,31^. Such work cites a variety of palaeoclimatic proxy evidence indicating a probably drier and cooler regime in the Eastern Mediterranean–Anatolian–Near East region in the period from the 13th to 10th centuries bc. However, this evidence is often only loosely placed in absolute temporal terms^20^ and provides a record of (at best) low-frequency climatic change. In the Hittite case, drought, famine, reduced harvests and concomitant reductions in workforce and military strength might be suggested as possible explanatory structures^32^, mirroring drought as the main threat to agriculture and food security in Anatolia in more recent history^33–36^. Several texts from the 13th century bc refer to apparent grain shortages or famine in Hittite lands. However, the interpretation of this material lacks detail and context^20,37^, whereas other critical assessments of the data from 13th century bc Hattusa do not necessarily indicate pending crisis^38^. Nonetheless, it may be observed that the Hittite Kingdom and especially its main centre, Hattusa, engaged in major landscape clearance^39^ (resulting in soil erosion) and specialized pastoralism^40^, and relied on higher-risk subsistence strategies dependent on water. Cereal agriculture both locally and from its periphery supplied the necessary large volumes of grain—attested in the early Hittite period by the discovery of a vast storage silo and subsequently via additional, smaller, dispersed silos^41^. In combination, these linked strategies may have increased production but amplified risk. As in several other early agrarian states, cereals formed a key subsistence and tax base^42^; in reverse, such states would have been vulnerable to serious and sustained threats to their cereal (and other crop) harvests and animal husbandry.

However, absent from such Late Bronze Age environmental resilience and sustainability assessments are nearly absolutely dated and highly resolved (that is, annual-scale) climate indicators for this region generally, and specifically from the Hittite administrative core in central Anatolia, capable of defining the nature of any critical climatic forcing potentially relevant to the collapse of the core Hittite administrative centre at multiple temporal scales. Gradual, low-frequency, shifts in climate whose amplitude does not completely alter the area’s bioclimatic system—that is, the shift attested in existing palaeoclimate archives for the period^1–4,30,31,43^—are less likely to undermine human strategies based around adaptation and resilience (through a wide variety of strategies of diversification, storage and social networking^5–8,44^). Similarly, one-year droughts (or comparable high-frequency challenges) are expected, particularly in a semi-arid region such as central Anatolia, where periodic and even regular droughts are anticipated as the main threat to agriculture^8,10,33–36,44^. Traditional farming practice (and storage strategies) in the greater Mediterranean region, and most agrarian cases, is adapted with the aim to be able to cope with one bad year^5,8–10,34,44^. What breaks the system—turning regular instances of food scarcity into famine and crisis—is multiple, consecutive harvest failures over two and especially more years^5,9,10,44^. On the basis of various records from the past several hundred years, such multi-year consecutive serious droughts leading to prolonged harvest reduction and failure (and so famine and associated threats from disease to violence) are rare but probably of potential historical relevance in central Anatolia^10,33–37,45,46^. Recognition of such critical episodes requires annually resolved data that facilitate multi-scalar (including high-frequency) climatic assessments. Here, high-resolution tree-ring records from central Anatolia enable us to examine climate change in the Late Bronze Age in decadal to multi-decadal generalities, but also with annual-scale and historical specificity. We use data derived from nearly absolutely placed tree-ring time series—both ring-width based and from ^13^C stable isotope analysis—to characterize an annually resolved climate record of moisture availability for the period around 1500–800 bc and investigate whether there was a catastrophic continuous multi-year drought episode of historical relevance across central Anatolia around 1200 bc.

**Extended Data Fig. 2:**
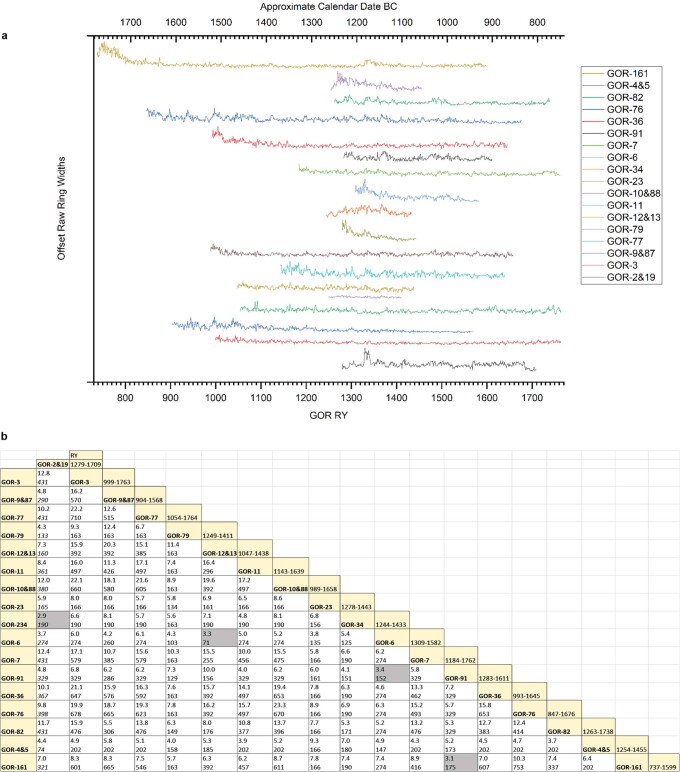
Gordion tree-rings series and crossdating grid. **a**, Raw (non-standardized) measured tree-ring series for the Gordion samples (all same scaling on Y axis) indicating the overall growth trends in each case. **b**, Crossdating grid for the Gordion tree-ring samples listing the Baillie-Pilcher t-scores (upper value) and length of overlap in rings/years (lower value) for each sample series versus the others from COFECHA^74^. Potentially satisfactory values where there is strong overlap (e.g. > 50-100 years) are t_BP_ ≥3.5 (unsatisfactory values are indicated by grey shading)—although in reality higher t_BP_-values are necessary for a secure match (see Methods). Higher values and especially t_BP_-scores >5-6 usually indicate a good crossdate. Here all samples exhibit at least several strong to very strong crossdates against a number of other samples in the chronology with long overlaps, demonstrating an overall robust chronology.

Juniper ( *Juniperus excelsa* and *Juniperus* *foetidissima*) timbers recovered from archaeological excavations at the site of Gordion in central Anatolia, about 230 km west of the Hittite capital Hattusa near the western frontier of the land of Hatti (the core kingdom of the Hittites), provide an annually resolved tree-ring chronology from around 1775–748 (±3) bc that traverses the end of the Late Bronze Age (Extended Data Fig. 2 and Methods). Twenty-three samples, representing around 18 different trees including the year 1200 bc, form a robust tree-ring chronology. Investigations of recent juniper populations in Anatolia and central Asia indicate that trees, especially from lower elevations (like those likely to be used at ancient Gordion), are susceptible to and exhibit reduced growth owing to decreases in late spring–summer precipitation^33,46–48^ (also critical to cereal production in Anatolia). Thus, examination of the tree-ring patterns in the Gordion tree-ring chronology and identification of episodes of substantially reduced growth increments (narrower tree rings)—after removing effects of age-related growth trends and stand dynamics from the series, and in the absence of any indications of fire or insect attack—probably indicates drier years, with extremes marking probable drought episodes critical to agricultural production and subsistence.

**Fig. 1:**
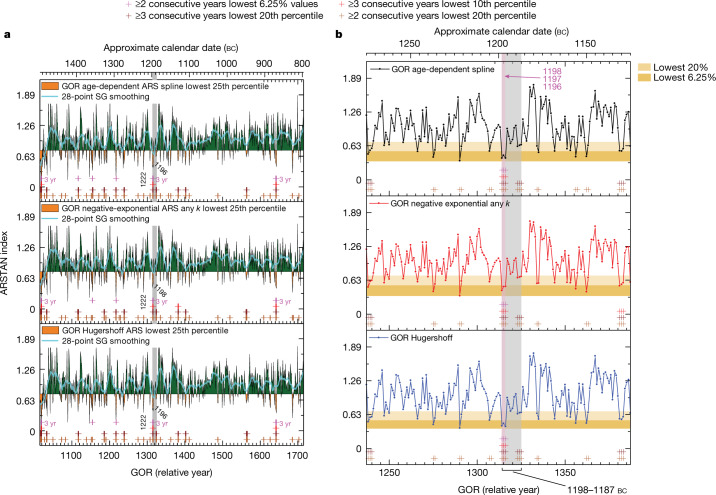
Proxies of drier to drought climate from three different detrending methods applied to the Gordion tree-ring dataset. **a**, Tree-ring record from 1497–797 bc (Methods). The driest 25% of years is shown in orange (other 75% of years in green shading); division indicated by black horizontal line; 28-point Savitzky–Golnay (SG) filter is shown. Instances of 2 or 3 consecutive dry to very dry years at various levels are indicated. There are 3 instances of the driest 6.25% of years occurring consecutively (1494–1492 bc, 1198–1196 bc and 871–869 bc). The grey bar indicates the 12-year period from 1198 to 1187 bc with 3 consecutive years from 1198–1196 bc in the lowest 6.25% of all years and with 6 or 7 years (50–58%) in the lowest 20% of values. GOR, Gordion. **b**, Close-up of the period 1275–1125 bc, showing annual ARSTAN (ARS) index values (Methods) highlighting those in the lowest 20% and the lowest 6.25% of values.

**Extended Data Fig. 3:**
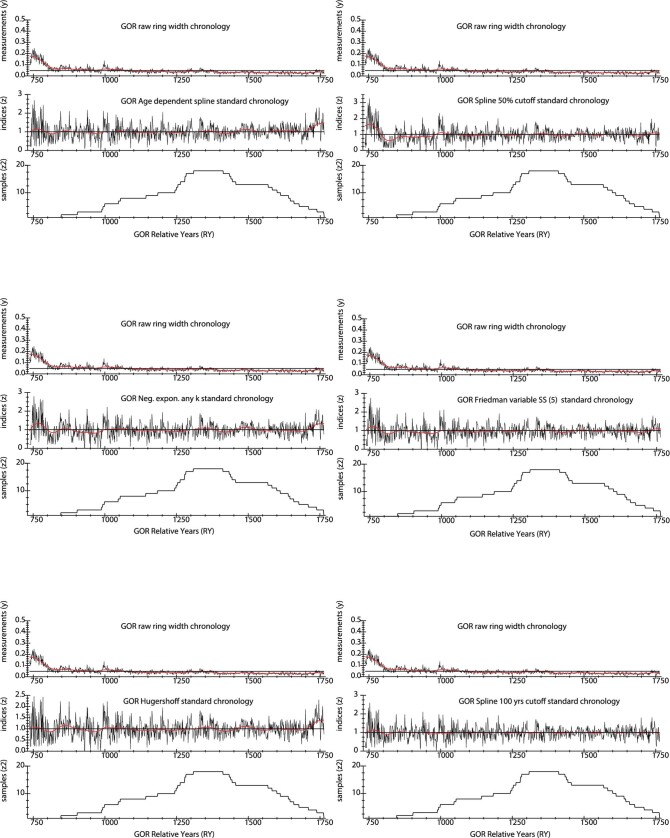
Ring-width measurements for the Gordion chronology and processing with ARSTAN^79,80^ and detrending to Standard Chronologies for the six different approaches used (see Fig. 1, Extended Data Figs. 5, 8a), each offering good correspondence with the data. Sample depth is shown under each figure element.

**Extended Data Fig. 4:**
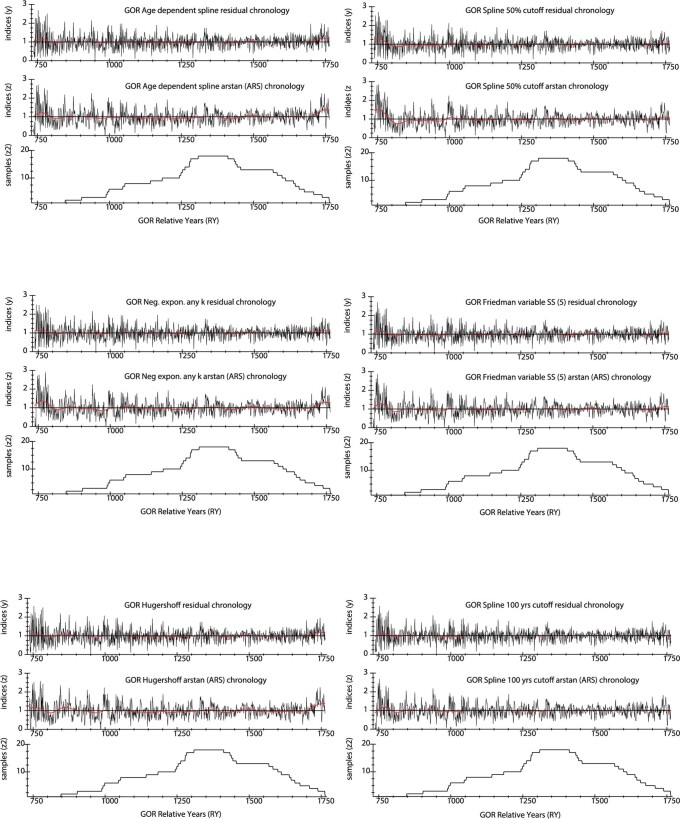
Residual and ARSTAN (ARS) chronologies derived from the six Standard Chronologies in Extended Data Fig. 3 using ARSTAN^79,80^. Sample depth is shown under each figure element.

**Extended Data Fig. 5:**
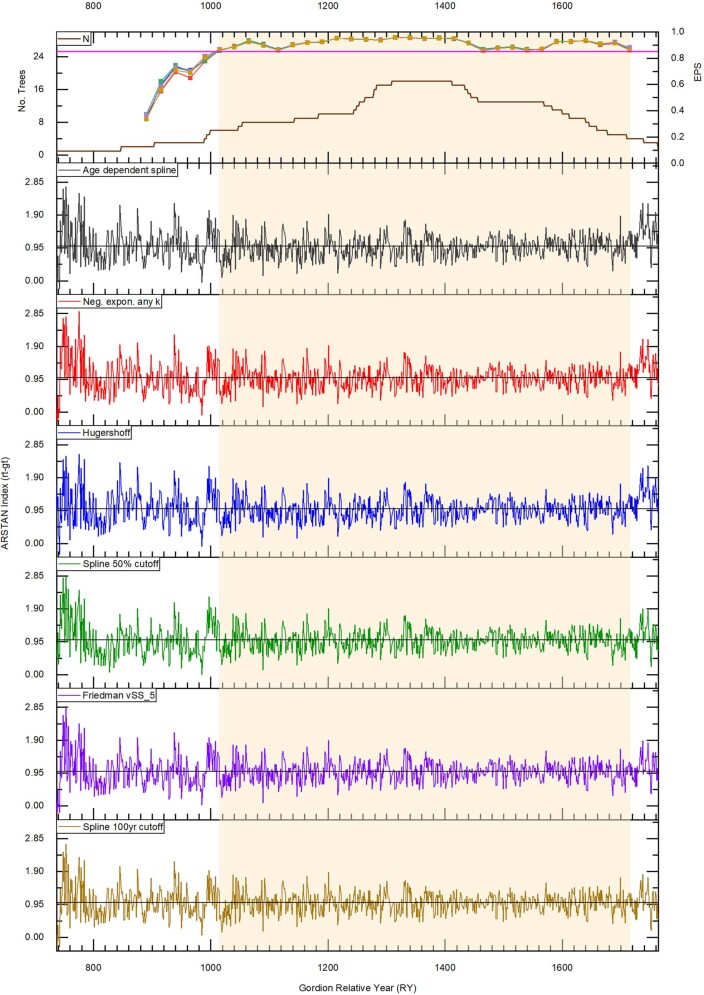
Sample number per year and Expressed Population Signal^86^ for a set of six ARSTAN (ARS) chronologies derived from the Gordion samples (see Extended Data Figs. 3, 4) using the ARSTAN software^79,80^ employing different standardization curves, each aimed at highlighting the likely underlying climate signal (see Methods). The shaded region indicates where the EPS values are ≥0.85. Note for the Hugershoff detrending one sample (GOR-3) receives the ‘itmax exceeded in amoeba’ warning—we used the age dependent spline for this sample.

**Extended Data Fig. 6:**
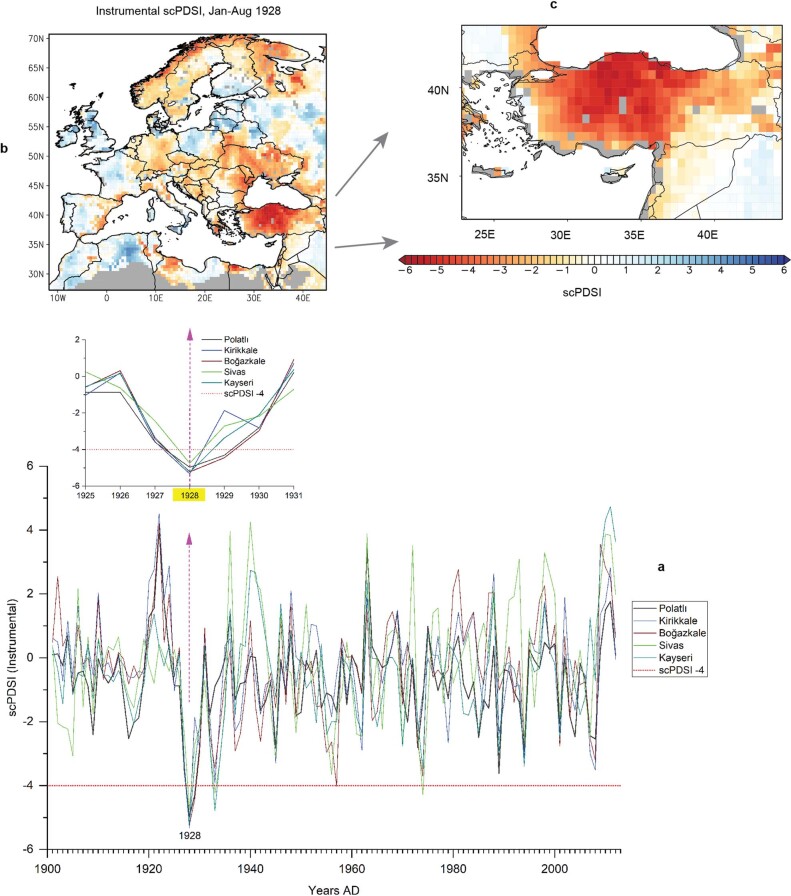
Modern Instrument-derived self-calibrating Palmer Drought Series Index (scPDSI) values and correlations ad 1901–2012 for January-August for central Anatolia from the Old World Drought Atlas (OWDA)^58^. **a**, Comparison of a west-east transect across the core Hittite region in central Anatolia from Polatlı (39.58N, 32.14E) to Kirikkale (39.85N, 33.5E) to Boğazkale (40.02N, 34.61E) (location of Hattusa) to Sivas (39.75N, 37.02E) and for a major center to the southeast (Kayseri) (38.72N, 35.48E) (see Extended Data Fig. 1). The scPDSI series from Polatlı has Pearson correlation coefficients (*r*) *=* 0.78, 0.7, 0.48, and 0.6 respectively; thus positive and suggesting a general regional pattern, except for Sivas in the east. **b**, Critically, the one extreme drought event in the dataset, centered 1928, occurs as a < scPDSI -4 event across all five loci, and **c**., is reconstructed across the entirety of central Anatolia, suggesting that extreme arid events recorded at Polatlı are likely regionally effective (see also Fig. 3 and Extended Data Fig. 7).

**Extended Data Fig. 7:**
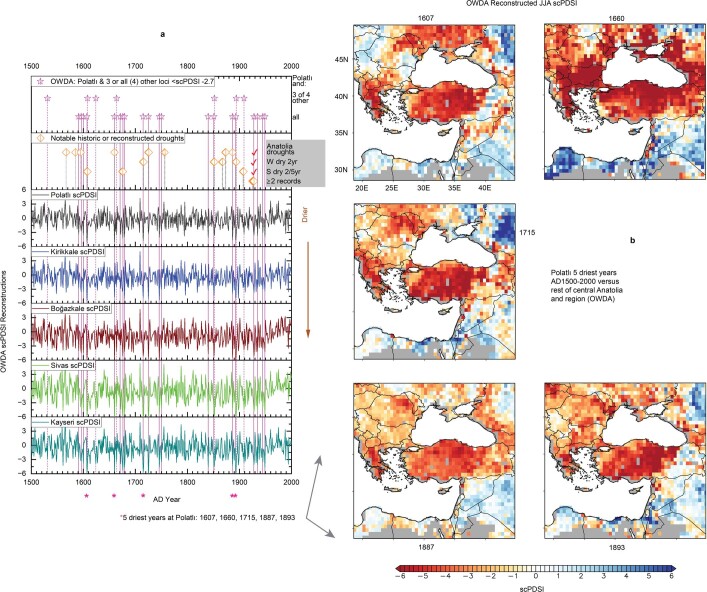
Tree-ring derived reconstructed self-calibrating Palmer Drought Series Index (scPDSI) values and correlations ad 1500–2000 (when there is reasonable data representation for Anatolia^35^) for June-July-August for central Anatolia from the Old World Drought Atlas (OWDA)^58^. **a**, Comparison of values from a west-east transect across the core Hittite region in central Anatolia as in Extended Data Fig. 6. Pearson correlation coefficients (*r*) with Polatlı (in vertical order shown) = 0.86, 0.85, 0.89 and 0.89 indicating a strong regionally coherent pattern. Instances where Polatlı and either 3 (of 4) or all 4 other locations have scPDSI less than −2.7 (value indicating lowest 6.25% of values at Polatlı) are indicated, as are some historically attested or reconstructed droughts starting ad 1567 (ref. ^46^ at Table 3), the reconstructed 2yr dry episodes for western Anatolia ad 1786-1930 (W dry 2yr) (ref. ^33^ at Table 6) and instances of noted occurrences of 2yrs or 5yrs of drought in southern Anatolia (S dry 2/5yr) since 1500^137^. All the five OWDA PDSI series record the same major arid episodes with **b**, showing that there is a strong wider regional expression across central Anatolia for the extreme arid events such as the five driest years in the Polatlı series in the OWDA dataset. In contrast, some of the other historically recorded droughts or those reconstructed for western or southern Anatolia do not correlate with especially dry conditions in one or more of the Polatlı, Kirikkale, Boğazkale, Sivas, and Kayseri series—events where the dashed grey bars do not correspond with the magenta lines in **a**—and so may not reflect serious drought expressed broadly across central Anatolia.

**Extended Data Fig. 8:**
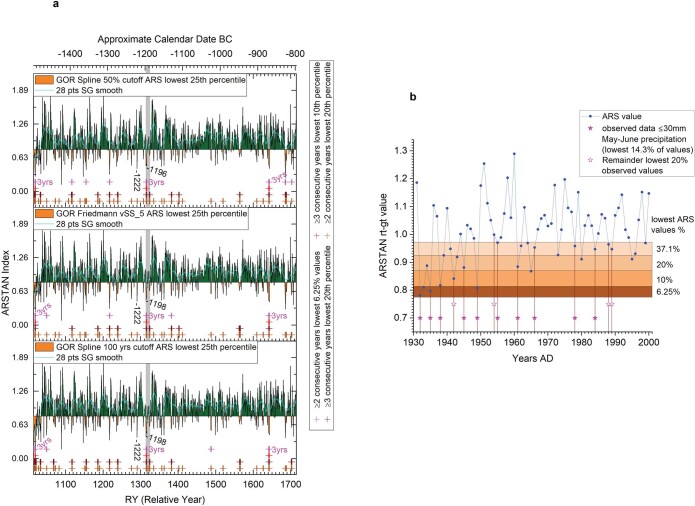
Additional ARSTAN (ARS) climate (moisture availability) proxies and comparison of ARS index values for a SW Anatolia juniper chronology versus observed low precipitation years. **a**, Three additional alternative versions of the Fig. 1 analyses. Drier calculated conditions (low index values) toward the bottom (compare with Fig. 1). See Fig. 1 for description of analysis. Grey bar indicates 12-year period ~1198–1187 bc with 3 consecutive years ~1198–1196 in lowest 6.25% of all years and with 7 or 8 years (58–67%) altogether in lowest 20% of values. **b**, ARS rt-gt values for three juniper tree-ring chronologies from SW Anatolia^87^, ad 1931–2000, versus the lowest observed precipitation totals for May-June, the period critical to annual precipitation response, from proximate meteorological stations^46^. The lowest (minimal) 37.1% of ARS values include the lowest 20% of observed precipitation values. The lowest 20% of ARS values include 50% of the lowest 20% of observed values and 7 of 9 (78%) in the period ad 1931–1961. The lowest 10% and 6.25% of ARS values are also indicated; with 5 of 7 (71%) of the former and 3 of 4 (75%) of the latter corresponding with years in the lowest 14.3% of observed years.

We investigated the Gordion chronology, detrending the individual tree-ring width series to derive a maximized climate signal, which we compared with modern analogous climate data for the central Anatolian region (Extended Data Figs. 1, 3–7 and Methods). Taking the lowest 25% of growth values in the Gordion chronology as indicating drier years, these events occur regularly between around 1497 and 797 bc (Fig. 1 and Extended Data Figs. 5 and 8a). For comparison, the Polatlı meteorological station, which offers the nearest modern climate analogue to ancient Gordion (17 km away), records less than 300 mm of annual precipitation for approximately 20% of the period from ad 1929 to ad 2009. Total annual precipitation of 300 mm is regarded as an approximate minimum threshold for a viable wheat harvest^44^. Only 6.25% of years from ad 1929 to ad 2009 recorded less than 250 mm of annual precipitation, less than the amount generally regarded as the minimum for cereal cultivation in the Near East, and an amount that would probably produce serious harvest reduction or failure^44,49^. Although regional precipitation values vary, the largely semi-arid central Anatolian region demonstrates overall congruent records or trends (Methods). In particular, recent and historical instances of extreme arid years are usually common to much of the greater central Anatolian or core Hittite region^33,35,36^ (Extended Data Figs. 6 and 7). Thus, if we regard the relative occurrence ratio of drier years in the recent Polatlı record as indicative for the broadly similar period of the drier later second millennium bc in central Anatolia^30^, and use the lowest 20% of values as indicating probable substantial reductions in the harvest, then the Gordion chronology contains only 80 or 85 out of 701 years (11.4%–12.1%) that are part of two or more consecutive such dry years. Between 1270 and 1135 bc, when we also have our best sample replication with 10–18 trees per year, there are only six such sets of years. Only 13 or 16 years (1.9%–2.3%) in total are part of two or more consecutive drought years with the lowest 6.25% of values, representing probable serious harvest reduction or failure. Between around 1270 and 1135 bc there is only one interval in these 135 years—1198–1196 bc—with two or more consecutive years in the lowest 6.25% of values (three consecutive years). Further, in the 12-year period from 1198 to 1187 bc, there were between 6 and 8 years (50–67%) in the lowest 20% of values (Fig. 1 and Extended Data Fig. 8a). Smoothing the data with a 28-point Savitzky–Golay filter representing an average human generational timeframe^50^, the period around this time represents either the driest or second-driest multi-year interval between 1400 and 1000 bc. This extremely dry interval stands out as a probably substantial climate challenge to food production and subsistence in central Anatolia that may have defeated normal strategies and storage provision in the Hittite administrative core. The dates—approximately 1198–1196 bc—are compatible with the historically derived timeframe of Hittite collapse and reorientation^4,11–13,16–23,32^, and lend an historical specificity that is usually lacking in general low-frequency arguments suggesting linkages between climate and history.

**Fig. 2:**
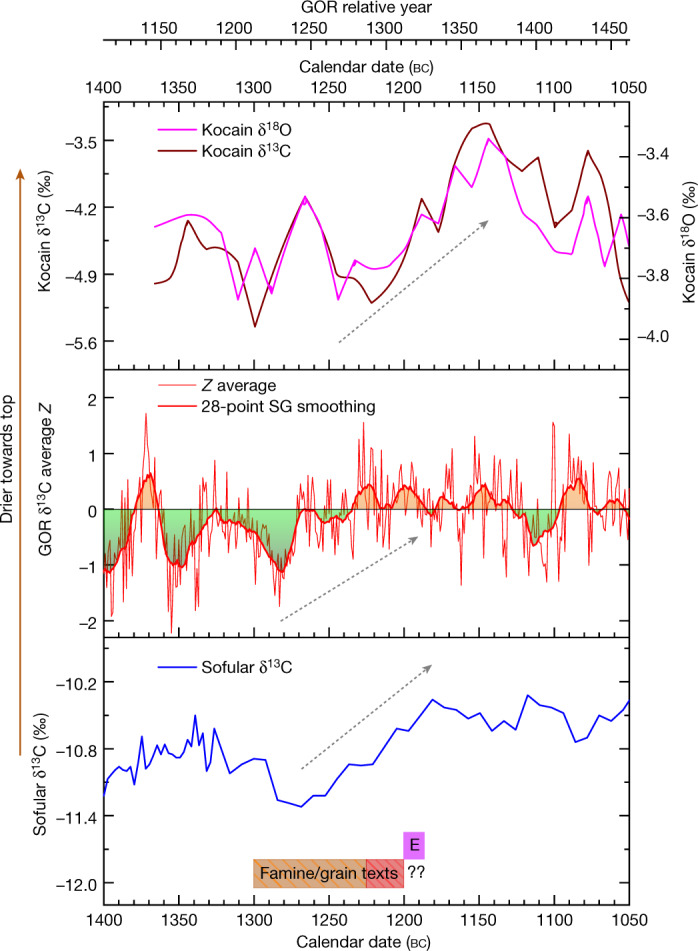
δ^13^C record from Gordion tree rings compared with δ^13^C and δ^18^O records from Sofular and Kocain Caves, Turkey, and the period referred to in texts mentioning famine or grain shortage in Hittite lands. δ^13^C and δ^18^O records from Sofular (bottom) and Kocain (top) Caves compared with the Gordion overall combined average *z* score δ^13^C time-series, δ^13^C_CorZ_, chronology (middle). The Gordion chronology is also represented smoothed with a 28-point Savitzky–Golnay filter (middle). *Z*-transformed values greater than zero (drier) are highlighted with orange shading above the line and contrasted with green shading for wetter conditions below the line. Dryness increases towards the top of the graph. Bottom, E indicates the End Hattusha–Hittite Kingdom period during the reign of Suppiluliuma II and before Ramesses III year 8. Bottom, out of 9 contemporary texts that refer to famine or grain shortage in Hittite lands^20,37^, 4 are from the 13th century bc, 3 are undated but also probably originate from the 13th century bc (orange shading), and 2 are from the end of the 13th century bc (red shaded area). The dashed arrows highlight indications of a drying trend in each record.

An alternative approach to recovering proxy moisture availability levels from tree rings at lower elevations in a semi-arid area such as central Anatolia is via ^13^C stable isotope analysis, with higher δ^13^C values usually indicating drier conditions. The δ^13^C is defined as [(*R*_sample_ − *R*_standard_)/*R*_standard_] × 10^3^, in which *R*_sample_ is the ratio of ^12^C to ^13^C in the unknown and *R*_standard_ is that same ratio in the internationally agreed-upon standard PeeDee Belemnite (PDB). The δ^13^C is driven by the gradient of CO_2_ between the atmosphere and the interior of the tree leaf, which is controlled predominantly by leaf stomata, the leaf apertures that control water loss and CO_2_ transport into the tree. Therefore, δ^13^C values fluctuate in response to changing moisture availability. We compare average *z*-transformed results (where positive values indicate higher δ^13^C) from a series of ^13^C measurements on α-cellulose extracted from tree rings of four of the Gordion trees with stable isotope records from the Sofular Cave (northwest Anatolia) and Kocain Cave (southwest Anatolia) for the period 1400–1050 bc (Fig. 2 and Methods). The data show a noticeable gradient towards drier conditions in the later 13th century bc, then probable consistently dry to very dry conditions as exhibited by the Gordion tree rings across the interval 1232–1192 bc with drier spikes from 1222 to 1221 bc and around 1195 bc, aligning closely with ring-width minima (Fig. 1 and Extended Data Fig. 8a). This period of drier gradient matches the timeframe of the ancient texts indicating grain shortages in Hittite lands^11,20,37^ and peaks around the time of the Hittite collapse, consistent with the tree-ring width analysis.

## Discussion

Critiques of many attempts to associate climate change with specific historical or archaeological shifts have focused on the often simplistic and reductionist logic, noting that there is frequently little consideration of how the climate shift is likely to have affected the relevant human subsistence and wider social, economic and political systems^6,7,51^. In several cases, the timeframes on one side or the other are not sufficiently highly resolved to enable a discussion of whether a specific correlation might exist—let alone whether such a specific correlation might in fact provide an element of causation^20,52^. Climate alone does not create or cause history. Rather, it is one of the forces comprising the context (*habitus*) in which human and other actors and vectors make decisions, interact, live lives and affect their surroundings. Humans, especially those living in semi-arid circumstances, expect both variable weather and year-to-year climate differences, and develop practical and physical adaptations (such as agricultural strategies, storage and water infrastructure) and social life (forms of networking) to create resilience against realistic and anticipated scenarios^5,8,34,44^.

As noted, the Hittite Empire and its core were accustomed to drought threat or stress. Evidence of military installations and political iconography point to efforts to manage territory and landscape^11,12^. Major water management infrastructure (such as dams and reservoirs) and their strong ritual associations point to efforts to control water resources and ameliorate deficits^53,54^. However, as observed for the medieval Middle East, “even well-organized regimes found it hard to cope with long periods (more than two years) of food shortages”^55^. Such periods were not anticipated. In Late Bronze Age Anatolia, although dry years and droughts were a regular feature, and even pairs of arid years generally (although not always) occurred at least once a generation, major droughts causing serious harvest failure in consecutive years were much rarer, probably occurring only once or at most twice in a century (Fig. 1 and Extended Data Fig. 8a). This is similar to observations from the second millennium ad^10,33,35,36,45,46^. In this context, the evidence of a serious drought and major harvest reduction in central Anatolia for three consecutive years between 1198 and 1196 bc, towards the end of a period from 1204 to 1192 bc according to tree-ring width data (Fig. 1) or from 1230 to 1192 bc according to the δ^13^C data (Fig. 2) indicating generally lower moisture, is probably an historically important episode that would have severely challenged existing adaptations and resilience strategies. Large land-locked centres and territories reliant on regional grain production and specialized animal husbandry (such as Hattusa and the core Hittite realm, along with the Empire’s overall subsistence and tax base infrastructure) may have been particularly vulnerable in such circumstances (despite various other loci being able to adapt and survive). Outside a few peripheral maritime or riparian locations, marshalling substantial long-distance shipments of bulk subsistence products—if available—would have been logistically impractical for central Anatolia given the over-land transport technology of this period (over-land pre-modern caravan routes of the region centred on items of low bulk and higher value per unit volume or weight^56^). Much of the Hittite heartland would have been effectively isolated and forced to survive on local resources, and, consequently, in crisis as these were progressively exhausted across one, then two, and finally three years of consecutive serious drought. Further, textual evidence hints that surrounding entities, and especially some of the maritime linked centres (such as Ugarit)—which had their own difficulties^20,37^—withheld possible grain shipments, in turn exacerbating crises in Anatolia. Intra-Anatolian conflicts may have been stimulated by struggles for key subsistence resources in and immediately following this period. Such circumstances would further stress underlying political, economic and social fault lines within the Hittite world that had apparently been building during the 13th century bc^21,23^, and around it, and also provide the context for disease outbreaks. Thus, we propose that these three years, between around 1198 and 1196 bc (and the period from 1198 to 1187 bc), may well mark and form a key part of the circumstances that precipitated the collapse of the Hittite Empire. However, we also must acknowledge that we lack the evidence to establish direct causation. It remains likely that this rare, disastrous climate episode from 1198 to 1196 bc tied in with (or enabled) other, probably human, forces that merit further examination. This drought event thus contributed to, but did not solely cause, the collapse and break-up of the Empire.

**Fig. 3:**
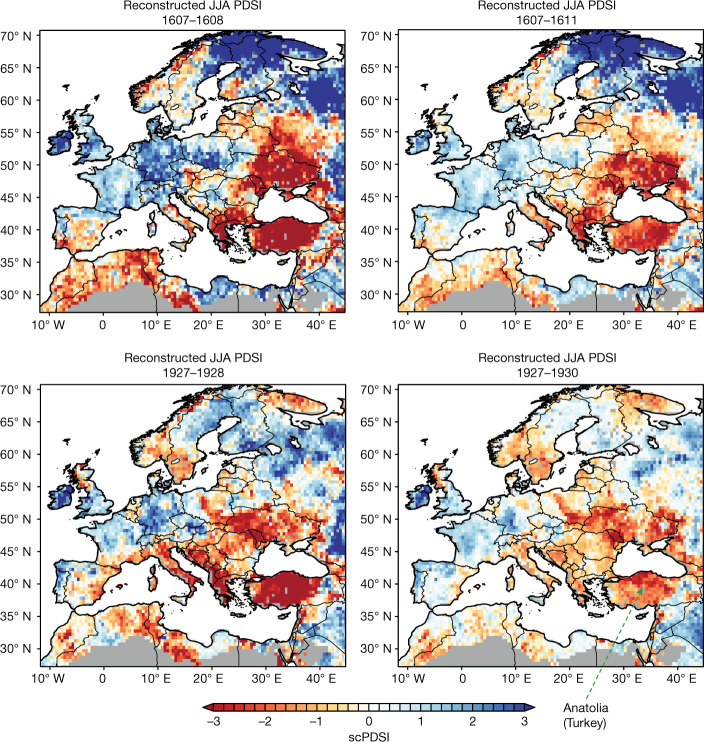
Reconstructed summer dryness levels across Europe and the Mediterranean region around the consecutive major drought years ad 1607–1608 and ad 1927–1928. Climate proxy data from the Old World Drought Atlas^58^ (http://drought.memphis.edu/OWDA/) derived from tree-ring measurements, showing self-calibrating summer (June–July–August (JJA)) Palmer Drought Severity Index (scPDSI) values (negative values indicate drier and positive values indicate wetter) across the Old World region during and around the consecutive major drought years ad 1607–1608 and ad 1927–1928 (2 consecutive years)^10^. Our study suggests that there were 3 consecutive drought years from 1198 to 1196 bc in Anatolia.

The Hittite collapse forms part of a wider set of changes occurring across the Old World around 1200 bc^1–4,17–20^. Climate alone was not the sole cause of these changes; very different histories are evident within the greater region^20,57^ (Methods). Nonetheless, if we consider the two instances of major back-to-back drought widely attested in Anatolia in the past seven centuries^10,36,45,46^, alongside data and analysis available from the tree-ring width proxy reconstructions in the Old World Drought Atlas^58^, we find that such severe drought tends to affect most of the area of the Hittite Empire, albeit with some variation in drought intensity (Fig. 3 and Extended Data Figs. 6 and 7). At the same time, as evident from these cases, other areas in the greater Mediterranean–Near East may well have experienced differing circumstances. Such strong differentials, with decisive adversity affecting an adjacent zone, may have helped to fuel population movements, reorientations of trade and political fragmentation, leading to new political alignments and configurations (and not just blanket collapse), and thus also form part of the explanation for the major regional reorientation in the early 12th century bc in conjunction with severe drought in Anatolia^20,57,59^.

Low-frequency climate change creates long-term general forcing conditions affecting human evolution and strategies of adaptation and resilience. However, it is the potentially critical vulnerability of established human systems to unexpected and consecutive multi-year extremes, with concomitant combinations of stresses, that can break and overwhelm established adaptations and resilience practices—and greatly amplify the effects of high-risk land management practices and degradative land use. This applies in history as well as the present in the face of current climate change. The probable multi-year major drought that we identify occurring from 1196 to 1198 bc in Anatolia offers a salient example.

## Methods

### Proxy climate data and history of climate and society

It is important to acknowledge that (1) all proxy climate reconstructions are both approximate and limited; and (2) the human dimensions of effects and responses to climate (and other) change are complicated with internal diversity and varying systems from bottom to top (of any hierarchy) within societies and networks of external connections affording plural possible trajectories^6,7^. Climate anomalies or changes by themselves do not cause human societies to rise or fall. They are part of the context within which individuals, groups, and larger social, economic, and political entities make decisions, act, and react^5–7,16,22,26,28,34,45,51,57^. As argued in the main text, multi-year (thus prolonged and extended) climate (or environmental) challenges^60^, such as unusual dryness in a semi-arid context (that is, drought), create circumstances that especially challenge existing systems. There is no simple correlation of crisis equalling collapse; indeed, in history there are few cases of real total collapse, rather, there is transformation (adaptation)—for example, the end or marked change of an existing political system or hierarchical model (such as the end of an empire or state)—to accommodate changing circumstances and usually with considerable continuity in many of the other, underlying, aspects of life^6,7,16,40,59,61^. The scale or harshness of the change (the ‘collapse’) in such transformations depends on many factors, from the severity of any climate driver to the constitution and history of the relevant society. In particular, for circumstances in which a ‘rigidity trap’ applies, as a previously successful, long-term system becomes entrenched and homogenous across a large area, there may be a harsh transformation^62^.

**Extended Data Fig. 9:**
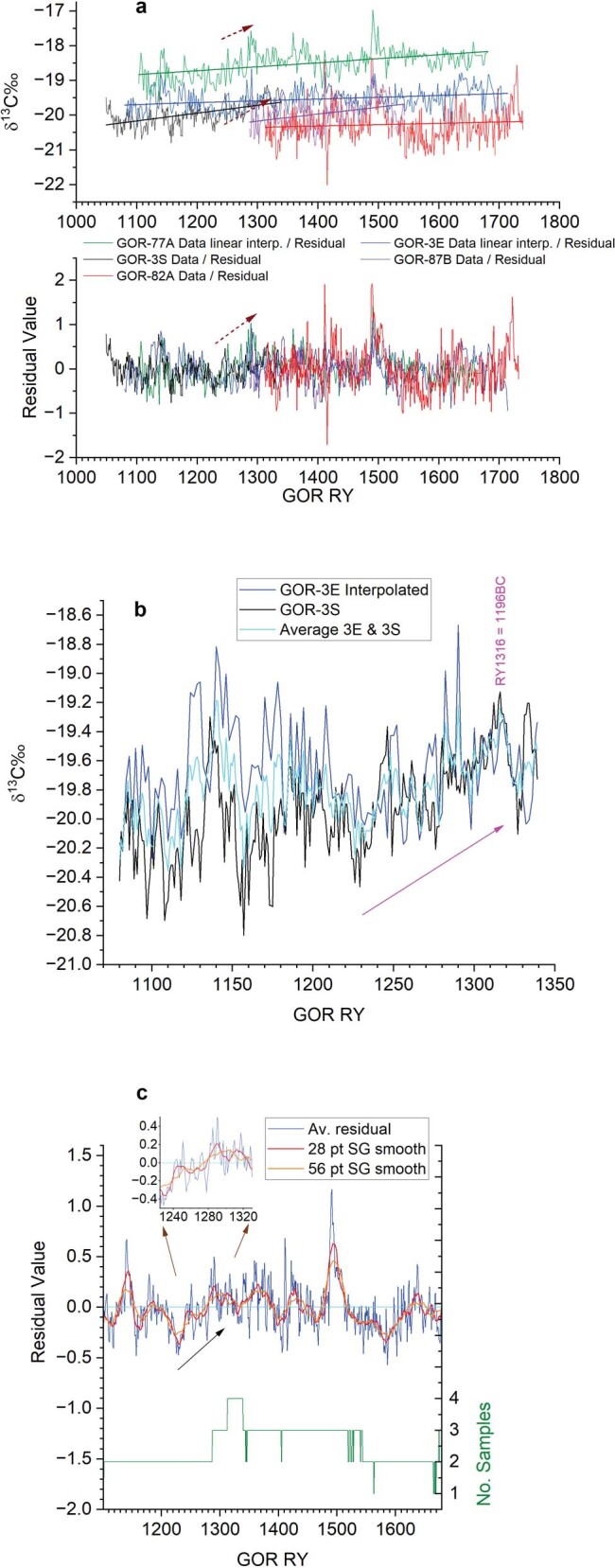
Gordion δ^13^C data, raw and residual values, and trends. **a**, Upper: plots of the δ^13^C data (with linear extrapolation between data points). The linear fit lines indicate small gradients towards less negative values with increasing tree age^101^. The arrows indicate a marked trend towards less negative values from ~GOR RY1230 to 1320. Lower: residual plots (from linear fits) removing different baselines and the age-trends. Gradient ~GOR RY 1230–1320 is evident. **b**, δ^13^C values from the two series on tree GOR-3. Similar baselines, and, despite noise, similar trend GOR RY1220–1320 (indicated by arrow). **c**, Average residual values (from **a**, lower) with 28 and 56 pt Savitzky-Golnay (SG) smoothing lines (one and two human generations^50^) for period mainly with ≥2 data per year. Trend to less negative values GOR RY1220–1320 is indicated (see also inset). The plot at the bottom indicates the number of data values per year.

**Extended Data Fig. 10:**
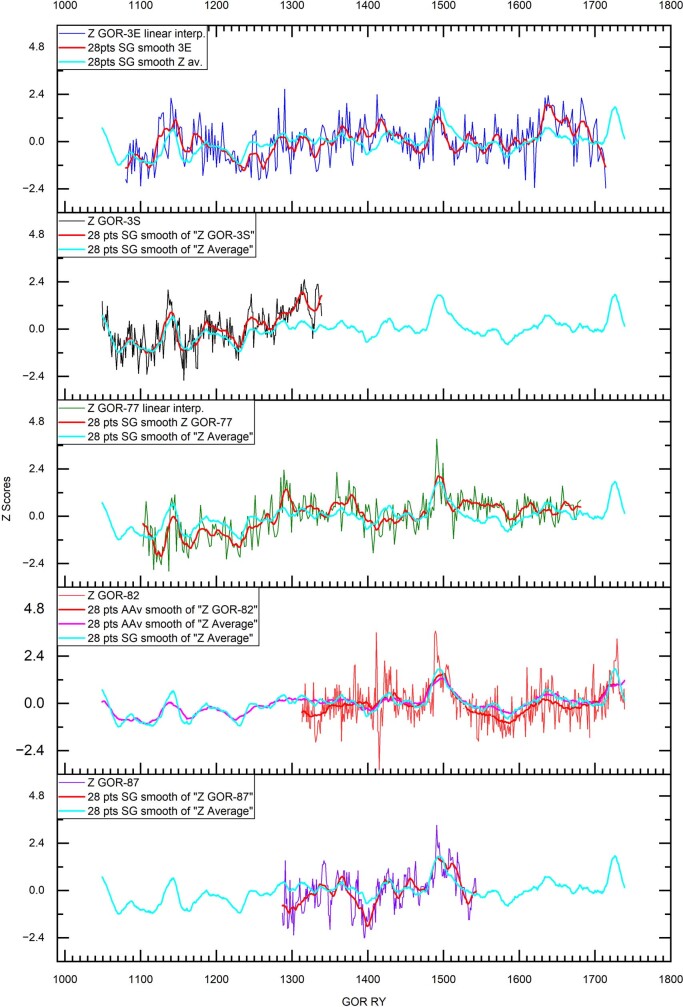
Indicative δ^13^C data and analysis from four Gordion trees (see SI, Table 2). Z scores for the five δ^13^C series comparing these via 28 pt Savitzky-Golnay (SG) smoothings (or 28 pt adjacent average smoothing in the case where the data are not all evenly spaced) versus the 28 pt SG smoothed curve of the average z values for the combined δ^13^C series (δ^13^C_CorZ_) (see Methods). The correlation coefficients between compared smoothed curves versus the smoothed average curve are GOR-3E (0.62), GOR-3S (0.9), GOR-77 (0.73), GOR-82 (0.80) and GOR-87 (0.78).

The Hittite case addressed in this Article engages with all these issues. We identify, but cannot exactly quantify, both (1) a period of increasing dryness (during the 13th century bc) followed by several decades of dry circumstances (late 13th to early 12th centuries bc—which is not necessarily the most extreme in the record but nevertheless a conspicuous trend) (Fig. 2 and Extended Data Figs. 9 and 10), and (2) within the latter dry period, a trio of consecutive years with unusually low tree-growth indicating very dry conditions that probably created extreme stress on subsistence agriculture and food storage strategies (Fig. 1 and Extended Data Fig. 8a) (see ‘Tree-ring data’ and ‘Stable isotope data’ below), which seems coincident with and part of the circumstances in which the large political entity of the Hittite Empire (probably falling into a rigidity trap context by this time) suffered a harsh transformation. At the same time, this collapse was a dynamic transformation, and many other aspects of society, economy and politics continued in the region (see further below)^20,22,26–29,40,57,59^.

### Tree-ring data

A tree-ring chronology comprising *Juniperus* sp. samples (*J. excelsa* and *J. foetidissima*) from archaeological contexts at Gordion, central Anatolia, and which were probably procured from the surrounding region, has previously been constructed using standard methods^63–65^ based on tree-ring width analysis and discussed in terms of locus, site context, and near-absolute dating^66–69^. These trees grew in the period before substantive human modification of the wider Gordion landscape (in the Iron Age to the Roman period^34,49,70,71^), probably on hillsides of lower to moderate elevations, and thus can be expected to reflect the area’s natural environment and climate. While there is no modern juniper population remaining in the Gordion region to act as a direct analogue to the archaeological material, recent studies of the same juniper species growing in neighbouring regions produce positive growth responses to greater spring–summer moisture availability and negative responses to increasing temperature^33,46–48,72^. We do not attempt to quantify proxy precipitation or moisture availability given the lack of a substantial modern analogue in the immediate region around Gordion. Both available modern analogue juniper trees and long-term meteorological (pre-1950s) data are absent. Instead, we seek to characterize indications of unusually dry conditions, both from instances of unusually narrow (reduced) tree-ring growth, consistent with modern occurrences of dry and/or hot conditions^33,46–48,72^, and via a shift in stable ^13^C values only plausibly explained by reduced moisture availability (see ‘Tree-ring data’ and ‘Stable isotope data’ below). Thus, we offer what we may term ‘moisture sensitive proxies’ and identify when these appear to indicate a multi-year unusually dry interval (see further below).

The Gordion juniper chronology runs from relative years 737–1764 or approximately 1775–748 ±3 bc (an alternative dating scheme would place the chronology approximately 3 years later or more recent^73^). Twenty-three *Juniperus* sp. samples, probably representing 18 trees include the year ~1200 bc (Extended Data Fig. 2). These were selected for the current project. Using the quality control COFECHA software (version 6.06P)^74^, the samples crossdate to form a robust chronology (Extended Data Fig. 2). All samples crossdate well with long (>100 years) overlaps with at least several other samples within the series set. The minimum acceptable Baillie–Pilcher crossdate *t*-value (*t*_BP_)^75^ employed in COFECHA is 3.5. For long (>100 years) crossdates this (somewhat arbitrary) value stands up well as an approximate threshold^76^. However, in reality, many years of dendrochronological work has shown that higher t_BP_ values are often necessary to produce secure crossdates^77,78^. Our Gordion series have a much higher mean *t*_BP_ value of 8.98 (and mean *t*_BP_ = 9.17, when four tree-ring series with instances of *t*_BP_ <3.5 against the rest of the chronology are excluded), with the average of all crossdated overlaps covering 304 years (and 309 years removing the 4 cases where *t*_BP_ <3.5). In the whole series only two overlaps (*n*) are <100 years (one at *n* = 74 years with *t*_BP_ = 4.4, and one *n* = 71 years with an unsatisfactory *t*_BP_ = 3.3). The series intercorrelation, measured as a Pearson correlation coefficient (*r*), is *r* = 0.632. Ring-width data employed are listed in Supplementary Table 1. COFECHA did not identify any errors in which all or part of a ring-width segment had higher correlation against the rest of the site chronology other than at the dated position. With the default COFECHA settings (segment length 50 years, lagged 25 years) COFECHA finds 29 outlier segments with low correlation but, if the segment length is increased as appropriate in a case like this with long series lengths and relatively complacent data^65,74^, then the number of flagged outliers reduces substantially, consistent with a robust chronology.

In order to derive a likely climate signal (moisture sensitive proxy) we used the ARSTAN software (version 49v1b_MRWE)^64,79,80^ to standardize the chronologies (Supplementary Table 1) by eliminating age-related growth trends and other possible non-climatic influences on tree-ring growth such as stand dynamics and local disturbances. Given the characteristics of the raw ring measurement profiles (Extended Data Fig. 2), which include a general negative exponential growth trend in most cases, some instances of initial suppressed growth, and generally long, largely complacent mature growth phases, we considered six detrending options (we did this to ensure or show that the unusually low tree-ring growth in the years ~1198–1196 bc is a consistent and robust observation across a reasonable ensemble of analytical approaches^81^). These comprised the following ARSTAN standardization curves: (1) the Melvin age-dependent spline^82^; (2) negative exponential curve (any *k*); (3) Hugershoff growth curve (except one sample, GOR-3, where warning that ‘itmax exceeded in amoeba’, and hence we used the age-dependent spline for this sample); (4) a 50-year spline with 50 per cent variance cut-off^83^; (5) the Friedman variable super smoother^84^ with mid *α* = 5 setting; and (6) a relatively stiff spline with a 100-year cut-off, given the form of the data and the research aim to investigate higher versus low-frequency variations (observed growth versus expected growth) (Extended Data Figs. 3–5). We employ approaches 1–3 in Fig. 1. The same analysis using the other three detrending versions (Extended Data Figs. 3–5) is shown in Extended Data Fig. 8a. All analyses produced similar results.

The data were combined into an overall chronology with a bi-weight robust mean including use of the weighted Rbar stabilization method^85^. This reduces the influence of extreme values within the series^79^. ARSTAN then produces three chronology versions, a Standard (STNDRD) detrended version, a Residual (RESID) version which reuses the Standard chronology after autoregressive modelling of the detrended series, and an ARSTAN (ARS) chronology which combines the pooled autoregression (persistence common and synchronous among a large proportion of tree-ring series and therefore probably climate related) into the RESID version and is anticipated to express the strongest climate signal possible^79^. We employed the ARSTAN chronology for our analyses showing the residual or difference (rt − gt, where rt = observed growth and gt = expected growth) ARSTAN chronology (Fig. 1, Extended Data Figs. 4, 5, 8). The mean inter-series correlation coefficient (Rbar) and the expressed population signal (EPS) were determined using 50-year moving windows with 25-year overlaps to assess the strength and reliability of the chronology in representing the original source population’s mean growth pattern over time (we assume that the juniper trees are probably from a similar source area, as they were cut and employed for the same specific construction project^67^, and exhibit high correlation, but this is an hypothesis). The EPS values for the analyses exceeded the arbitrary but commonly cited threshold value of 0.85 regarded as suitable for dendroclimatological purposes^86^ between relative years 1015–1715 (~1497–797 bc) (Extended Data Fig. 5), and this is the period employed in our analyses (Fig. 1, Extended Data Fig. 8a). In our study, given the absence of available recent juniper data in the immediate region, it is not possible to quantify the relationship between relevant narrowest tree rings and then the detrended ARSTAN values per year (for juniper in the Gordion area) versus precipitation. Nonetheless, as a reasonable analogue, we might consider the relationships between juniper in Southwest Anatolia versus instrumental May–June precipitation (the critical months for a precipitation proxy in this region)^46^. Here, ring-width data are available for three juniper chronologies^87^ (https://www.ncei.noaa.gov/access/paleo-search/study/6301, accessed August 2022). We can compare ARSTAN modelling of these using the Melvin age-dependent spline^82^ (see Extended Data Fig. 8b). Observed meteorological data 1931–2000 find 10 years (14.3%) with ≤30 mm May–June precipitation (figure 2 in ref. ^46^). All are in the tree-ring chronology’s lowest 37.1% of ARS values, with 6 of these 10 years in the lowest 12.9% of ARS values and 4 of the lowest 5 ARS values (7.1%) matching against these years (and the exception, 1933, immediately follows one of the top-10 driest years in both the meteorological records and ARS identified years, and so might be seen as related to the preceding very dry year). All but one of the lowest 20% of observed years (92.9%) are also in the lowest 37.1% of the ARS values. The single driest year, 1935, yields the second lowest ARS value. Thus, while in no way an entirely direct relationship, we might reasonably conclude that the occurrences of groups of values in the lowest quintile, and then lowest 10% of values, and especially the suggested lowest 6.25% of ARS values in the Gordion juniper series, and their comparison in the main text against the Polatlı meteorological data^34^ probably (and qualitatively) indicates drier to very dry years. When identifying the lowest 6.25%, 10% and 20% of values, if more than one year had the threshold value, then the set included was reduced to be <6.25%, <10% and <20%.

Despite some variations, recent meteorological records demonstrate similar and largely coherent precipitation patterns for the Central Anatolia region (much of the core Hittite area) and for the south-central Black Sea region close/relevant to Hattusa^88–91^, with all falling into the same Continental Central Anatolia (CCAN) precipitation region^88,89^ (the approximate CCAN region is shown in Extended Data Fig. 1); the same precipitation shape classes, C or D, and magnitude classes, 1 (lowest precipitation values) or (one case only) 2 (second lowest values), and, overall, the inland composite precipitation regime regions as identified in ref. ^90^ (the combined approximate area of the two main composite inland precipitation regimes identified in ref. ^90^ at figure 8 are indicated in Extended Data Fig. 1). Instrumental (ad 1901–2012) and tree-ring derived Palmer Drought Series Index values ad 1500–2012^35,58^, further attest to this general coherence across much of central Anatolia, and especially for years of marked low precipitation (Fig. 3 and Extended Data Figs. 6 and 7). Thus it is reasonable to assume that instances of severe/extreme drought in the Polatlı–Gordion area, as indicated by the tree-ring record, are likely to translate as arid to very arid conditions more widely across much of central Anatolia (whether the meteorologically broadly similar and coherent Continental Central Anatolia region^88,89^, or the Central Anatolia and south-central Black Sea regions, or overall simplified inland precipitation regime regions, outlined in ref. ^90^), that includes Polatlı–Gordion, Hattusa–Boğazkale, and the other loci employed in Extended Data Figs. 6 and 7. The data for grid points approximating Polatlı, Kirikkale, Boğazkale, Sivas and Kayseri in Extended Data Figs. 6 and 7 were extracted from ref. ^58^. Hence, despite some intra-regional variation across distances of several hundred kilometers, we argue that use of the (unique) tree-ring series available from juniper trees that grew around the Gordion region (probably within ~30–50 km or so of the site, but perhaps even from a wider zone in central Anatolia^34,49,67^), before their use^34,49,66–68^, provide insight into major drought events that widely affected much of central Anatolia, including the region around the Hittite capital of Hattusa, and the core Hittite region and subsistence base.

Figure 3 and Extended Data Figs. 6 and 7 use data from The Old World Drought Atlas^58^ which are archived and freely available from the National Centers for Environmental Information (NCEI) at the National Oceanic and Atmospheric Administration (NOAA) (https://www.ncdc.noaa.gov/data-access/paleoclimatology-data). To select and represent these data we used the Tree-Ring Drought Atlas Portal^92^ (freely available and open-access) located at http://drought.memphis.edu/ which facilitates analysis of the gridded reconstructions (last accessed November 2022).

### Stable isotope data

The stable ^13^C isotope ratio of plant material is a reflection of the ratio of leaf external to internal CO_2_ concentrations (*c*_i_/*c*_a_) during growth. The *c*_i_/*c*_a_ ratio is primarily controlled by stomatal conductance, the aperture of pores on the leaf surface allowing for the exchange of CO_2_ and water vapour into and out of the leaf. During dry periods, plants close stomata to avoid water loss and hydraulic damage to plant tissue. Therefore, times of limited water availability are recorded in the cellulose of wood and, when combined with dendrochronologically dated tree rings, can be used as a first approximation of environmental drought history^93–97^. Previous isotope work on tree rings of *Juniperus* sp. and other conifer species in Anatolia identified a moisture availability signal^98,99^. To provide additional support for the drought hypothesis suggested in this study we investigated the ^13^C/^12^C isotope ratios of wood from four trees from the Gordion chronology: GOR-3 (two sections of the same tree but different parts and collected at different times), GOR-77, GOR-82 and GOR-87. This limited sample material was used to test whether there was a consistent indication of drier conditions around the period identified from the tree-ring width analysis, rather than production of a comprehensive, detailed, long-term stable ^13^C based climatic record. Wood sections whose tree rings overlapped the dated time period of interest, had sufficient mass for isotope analysis and were most accessible for dissection, were dissected using a steel blade under a binocular microscope. Possible juvenile tree rings were avoided, with sampling starting from 50 years (GOR-82), 59 years (GOR-77), 113 years (GOR-3) to 383 years (GOR-87) after pith or start of sample series. Where possible (in sections with sufficiently wide ring widths and available material) samples were dissected as single, annual, tree rings within the time period of the study. However, this was not always possible due to instances of very narrow tree rings and especially in cases of wedging tree rings or locally absent rings that reduced available material and practical sampling—in such cases the smallest number of practical tree rings was sampled. Whole annual ring samples were used (no division of early and late wood), whether for single tree rings where possible, or a multi-year group in other cases. For two series every second tree-ring was analysed. The samples were cut into small slivers with Soxhlet extraction used to remove labile carbon and then processed to α-cellulose in separate Teflon bags adapting a modified Jayme–Wise procedure^100^.

The carbon isotope ratio (δ^13^C versus Vienna Pee Dee Belemnite (VPDB)) data were obtained at the Cornell University Stable Isotope Laboratory using an isotope ratio mass spectrometer (Delta V, Thermo Scientific) interfaced with a high temperature pyrolysis analyser (TCEA, Thermo Scientific). The within-run isotopic precision of the methodology using quality control standards at the laboratory is ≤0.2‰ for carbon. The dataset employed from samples GOR-3S, 3E, 77A, 82A, and 87B is listed in Supplementary Table 2 (five sets, two from different samples from the same tree) and are shown in Extended Data Fig. 9. All data acquired are shown except for (1) the GOR-87 series was ended to exclude some data well after the time period of interest and (2) the GOR-87 series is shown from RY1287 as data were corrupted during the times preceding these data. In cases where a measurement was made on multiple tree rings, this value was applied to all of these tree rings; where data were collected on every second tree ring, linear extrapolation was used to create annual data for the comparisons reported. Since each individual tree derives from a unique microclimate, we do not expect them to have the same carbon isotope ratio. The exceptions are the two series from different elements of GOR-3. These show variation but a similar baseline level and both show rising levels relative year ~1230–1320 (~1282–1192 bc) to a consistent peak ~relative year 1316 (~1196 bc) (Extended Data Fig. 9). The important observation across the sets of values concern where there is a pattern of similar divergences in the trends recorded around the same times across several of the individuals, such as relative year ~1230–1320 (GOR-77A, 3E, 3S, 87B) (Extended Data Fig. 9). This strongly suggests each tree was experiencing drier conditions, even though they have different baselines. There appears to be evidence of a small trend to less negative ^13^C values with increasing tree age (Extended Data Fig. 9a, lower) consistent with observations of small age-related trends as recently quantified for a set of *Pinus sylvestris* L. trees from northern Fennoscandia^101^. However, the magnitude of this trend is much smaller than the diversions we have observed to indicate changes to drier conditions in this study. In particular, the key observation from Extended Data Fig. 9 is a general trend to less negative ^13^C values in the period from around GOR RY1230 to 1320.

The ^13^C data in Supplementary Table 2, and the different baselines and variations evident (the average value (±s.d.) from all data is –19.68‰ (±0.73‰)) correspond with other more recent time series of ^13^C on juniper from Anatolia (for example, figure 2 in ref. ^99^). The different baselines from different trees suggest that using and averaging the raw δ^13^C values is not appropriate. Instead, since there appear to be indications of several common trends, we consider the data in terms of (normalized) *z*-transformed values for each individual δ^13^C series (as in ref. ^99^). The trajectories of the individual *z*-transformed δ^13^C series exhibit a similar overall trend and were averaged into one mean site chronology, δ^13^C_CorZ_. We then applied a 28-point Savitzky–Golnay (SG) smoothing curve to δ^13^C_CorZ_ (with the 28-point (28-year) smoothing chosen as representative of average human generation timescale in pre-modern times^50^). We compare 28-point Savitzky–Golnay smoothing curves constructed from the individual data series versus this average (except in the case of GOR-82 where a 28-point adjacent average smoothing was applied as the data in this series are not all evenly spaced) (Extended Data Fig. 10). The smoothed individual series all correspond well with the smoothed δ^13^C_CorZ_ curve (see Extended Data Fig. 10) offering correlation coefficients against the smoothed δ^13^C_CorZ_ curve of GOR-3E (0.62), GOR-3S (0.9), GOR-77 (0.73), GOR-82 (0.80) and GOR-87 (0.78). The δ^13^C_CorZ_ curve values from Extended Data Fig. 10 are thus employed as representative or indicative of general moisture availability in Fig. 2. We highlight that this analysis is presented in support of the ring-width analysis and not as a pure stand-alone climate proxy. The number of separate trees employed is low. The focus of our investigation is merely to ascertain whether tree-ring stable carbon values indicate likely drier conditions (in relative terms irrespective of possible age-related and other trends) consistent with the tree-ring width analysis. The data and analysis we report indicate a relatively moderate (less arid) period from the mid-14th century bc to the earlier 13th century bc (green shaded areas) before a trend to noticeably drier conditions in the later 13th to initial 12th century bc (orange shaded areas) and then generally drier conditions from then until the late 12th century bc (Fig. 2). Outside the period of focus in this study, it is also noticeable that four trees indicate another arid episode in the record in the late-to-end 1020s bc (GOR RY1489–1494). There is a two-year consecutive lower growth interval GOR RY1487–1488, which may be associated (Fig. 1 and Extended Data Fig. 8a), but the tree-ring width response is neither as substantial nor clearly indicated as the GOR RY1314–1316 or ~1198–1196 bc case. The stable δ^13^C data from Sofular Cave shown in Fig. 2 are from ref. ^102^; the stable δ^13^C and δ^18^O data from Kocain Cave in Fig. 2 are from ref. ^43^. While not of the same calendar-scale resolution, both these cave speleothem records offer complementary and consistent indications of drier conditions towards and around 1200 bc (as indicated by the arrows in Fig. 2).

### Hittite and ancient Near Eastern history, geography and chronology

Hittite and surrounding ancient Near Eastern history and approximate geography are taken from refs. ^4,11–15,21,23–26,29,32^. Additional sources, regarding Hittite history, Empire, economy and sociopolitical structures, as well as the environmental and landscape contexts ~1200 bc, include refs. ^103–110^. The suggestion of drought as a, or the, possible cause of the Late Bronze Age collapse has a long history, first as a hypothesis; more recently it has been proposed as a century to multi-century-scale dry and cooler episode^1–4,30,31,111–116^. A variety of textual sources refer to grain shortages or to famine variously dated somewhere in the 13th century bc. Some more specifically date to the period of the late 13th century bc and around 1200 bc, and there are complementary indications of higher grain prices potentially consistent with shortages or supply problems^4,20,37,103,114,117,118^. What has been absent is evidence of a specific but potentially historically transformative multi-year severe drought. However, at the same time, while many studies concentrate on the widespread evidence for collapse in the period around and following ~1200 bc^4,17–19,109,114^, it is important to observe that, while widespread, collapse was by no means universal, and, at a number of sites (and areas), there is rather evidence of continuation, reorientation or even development during this time, and differential site and regional impacts and trajectories are observed^20,26,28,38,57,59,117,119–122^. In the case of the Hittites, in particular, it is the Hittite Empire and its central administration and the site of Hattusa (capital and religious centre or core), especially, that collapses (ending also the primary textual history available from the Hittite world until that time). Elements of the former Hittite world, smaller successor states in some cases, and some smaller sites in less challenged environmental settings even relatively proximate to Hattusa, like Çadır Höyük ~70 km distant, adapted and continued. Some other major sites, like Kaymakçı, show a marked decline from ~1200 bc, but perhaps include a final Late Bronze Age or Early Iron Age phase before abandonment^123^. As observed by ref. ^122^: “depending on one’s geographic location … the end of the Hittite Empire might have felt more like a bump or a bang”. As noted earlier, ‘collapse’ is thus rather best viewed as a form of transformation and so resilience.

Dates for Hittite kings (for example, accession of Suppiluliuma II ~1207 bc) employ the (Mesopotamian) Middle Chronology (as ref. ^11^). Both recent historical analysis, and dendrochronology combined with ^14^C analysis, support a Middle Chronology date range for Mesopotamia (and thence associated groups like the Hittites)^69,124–126^. The terminus ante quem for the end, collapse and destruction of the Hittite Empire is the inscription at Medinet Habu in Egypt dated to the 8th year of Ramesses III, which lists *Hatti* (the Hittites) among those entities destroyed or swept away by the Sea Peoples^17,127^. This is assuming the strict historical accuracy of what is largely a highly political text presenting and celebrating the success and victory of Ramesses III, alone, in repelling what he describes as, until then, an overwhelming tide of change—the account contains several likely issues regarding its veracity when critiqued in detail^127^. In scholarship over the past several decades dates for the accession of Ramesses III were usually placed in the mid-1180s bc (for example, 1187 bc^128^ or 1184 bc^129^), and so his year 8 somewhere 1180–1177 bc, based around the fixed point of a date of 1279 bc for the accession of Ramesses II. However, recent work on the historical evidence^130–132^, compatible with detailed radiocarbon assessment^133^, instead suggests dates 11 years earlier: thus Ramesses III’s year 8 would rather be placed at 1188 bc (and the accession of Ramesses II at 1290 bc). Other dates or last attested dates for Late Bronze Age sites in northern Syria that suffer destructions are in a similar range. For examples: (1) the destruction of the major coastal centre of Ugarit has been argued to lie between ~1193 and 1186 bc based on a synchronism with Bay, the Egyptian chancellor under Seti II but executed in the 5th year of Siptah—though these dates move ~11 years older if the earlier dates for Ramesses II and III noted above apply and strictly they form a terminus post quem rather than a date^20,118^; or (2) the site of Emar inland on the Euphrates was destroyed in the second year of the Babylonian king Melišipak (or Melišihu) ~1185 bc (Middle Chronology)^118,134–136^. The revised dates, with Ramesses III Year 8 placed ~1188 bc, and destructions, and mentions of grain shortages and famine, in a range from ~1200 to mid-1180s bc potentially offer a better fit with the dendro-^14^C dated severe, multi-year drought ~1198–1196 ±3 bc—and thus the Middle Chronology dates for the last Hittite king Suppiluliuma II—than the alternative lower Egyptian or Mesopotamian dates. (The lower dates would place, for example, the collapse of the Hittites nearly 20 years after the serious drought, rather than, as the former, around or shortly after ~1198–1196 ± 3 bc).

### Reporting summary

Further information on research design is available in the Nature Portfolio Reporting Summary linked to this article.

## Online content

Any methods, additional references, Nature Portfolio reporting summaries, source data, extended data, supplementary information, acknowledgements, peer review information; details of author contributions and competing interests; and statements of data and code availability are available at 10.1038/s41586-022-05693-y.

## Supplementary information

Supplementary Tables This file contains Supplementary Tables 1 and 2

Reporting Summary

## Data Availability

All of the data that support the findings of this study are available in the main text or Supplementary Information.
