# Supplementary material for: Severe multi-year drought coincident with Hittite collapse around 1198–1196 bc
Source: Nature. 2023 Feb 8;614(7949):719–24. doi: 10.1038/s41586-022-05693-y (PMC9946833; doi:10.1038/s41586-022-05693-y)
Supplement: Supplementary file 1 — This file contains Supplementary Tables 1 and 2 [file 41586_2022_5693_MOESM1_ESM.pdf]

nature portfolio

https://doi.org/10.1038/s41586-022-05693-y

Supplementary information

Severe multi-year drought coincident with Hittite collapse around 1198–1196 BC

In the format provided by the authors and unedited

Supplementary Information, Table 1|Tree-ring width data for the Gordion (GOR) samples analysed in this paper in units of 0.01mm in Tucson (.rwl) format.

Tree-ring width measurements employed for the following samples/trees from the Midas Mound (MM) tumulus, Gordion, and Kizlarkaya tumuli near Gordion, Turkey[66-68]. Samples of Juniperus sp. (either *J. excelsa* or *J. foetidissima*). Site elevation ~700m. Data in 1/100ths of a mm (0.01).

GOR-2 & GOR-19 = 500219MM
GOR-3 = 500030MM
GOR-9 & 87 = 500987MM
GOR-77 = 500771MM
GOR-79 = 500790MM
GOR-12 & 13 = 500123MM
GOR-11 = 500110MM
GOR-10 & 88 = 500108MM
GOR-23 = 500231MM
GOR-234 = 100000MM
GOR-6 = 500060MM
GOR-7 = 500783MM
GOR-91 = 500911MM
GOR-36 = 500360MM
GOR-76 = 500762MM
GOR-82 = 500082MM
GOR-4 & 5 = 500050
GOR-161 = 600610

Data for GOR-2ABCD, GOR-3E and GOR-161 have been previously submitted to the International Tree Ring Database: https://www.ncei.noaa.gov/access/paleo-search/study/3802

Data below amalgamate GOR-2 and GOR-19 (same tree).
Data for GOR-3 combines other GOR-3 samples.

500219 1 Gordion floating - Midas Mound tumulus, Kizlarkaya JUSP Relative:
500219 2 Turkey J.excelsa+foet. 700M 3938 03000
500219 3 Peter I. Kuniholm Maryanne Newton Bryant Bannister Jeffrey S. Dean Carol Griggs Jennifer Watkins Brita Lorentzen Sturt W. Manning

500219MM1279	30									
500219MM1280	18	71	27	46	46	34	44	25	22	23
500219MM1290	29	29	26	34	40	38	80	75	75	66
500219MM1300	67	60	58	61	77	97	73	66	67	63
500219MM1310	41	50	41	57	57	61	51	63	57	57
500219MM1320	54	71	61	48	43	40	61	58	82	122
500219MM1330	216	175	170	182	103	108	173	151	196	156
500219MM1340	138	125	95	62	63	66	70	73	76	80
500219MM1350	83	52	73	73	76	93	66	82	114	88
500219MM1360	113	86	68	71	96	100	84	94	63	82
500219MM1370	89	66	85	73	66	49	46	37	52	49

500219MM1380	39	34	35	50	57	69	80	62	92	80
500219MM1390	76	90	78	49	67	77	55	41	56	52
500219MM1400	70	42	35	38	48	43	47	53	67	73
500219MM1410	67	73	29	79	97	102	90	110	69	110
500219MM1420	55	73	66	65	59	76	85	75	71	54
500219MM1430	60	46	39	53	53	49	55	69	48	44
500219MM1440	55	58	63	61	46	49	39	55	43	50
500219MM1450	37	53	48	44	52	64	66	61	65	48
500219MM1460	69	52	46	52	56	57	57	72	60	68
500219MM1470	82	83	73	64	54	93	93	92	93	88
500219MM1480	98	42	76	81	78	94	83	50	51	52
500219MM1490	48	62	75	66	71	66	87	74	94	54
500219MM1500	60	88	92	99	65	76	58	83	75	77
500219MM1510	61	82	68	86	83	64	57	70	70	42
500219MM1520	60	51	65	60	27	29	28	40	27	35
500219MM1530	45	38	51	41	54	36	55	55	56	41
500219MM1540	44	40	51	53	49	58	45	39	54	55
500219MM1550	59	65	57	48	63	41	53	46	53	42
500219MM1560	59	46	38	26	40	45	45	67	7	88
500219MM1570	91	90	99	76	53	68	65	64	73	51
500219MM1580	72	69	76	83	50	62	66	60	59	61
500219MM1590	52	57	58	83	49	67	48	57	76	69
500219MM1600	84	56	75	56	51	75	53	39	59	47
500219MM1610	49	83	81	76	56	75	77	88	69	87
500219MM1620	57	70	63	66	54	66	49	57	73	70
500219MM1630	97	67	64	79	67	100	71	53	64	85
500219MM1640	59	59	49	32	62	60	68	72	46	72
500219MM1650	48	61	59	58	54	45	61	89	51	74
500219MM1660	79	84	69	71	58	91	76	83	79	88
500219MM1670	87	62	68	78	68	72	57	61	58	54
500219MM1680	77	93	77	84	39	23	12	39	25	18
500219MM1690	30	24	24	24	29	11	3	14	3	12
500219MM1700	8	1	16	17	43	29	5	4	19	18
500219MM1710	-9999									
500030MM999	50									
500030MM1000	70	68	77	54	70	79	63	50	94	73
500030MM1010	27	57	69	59	61	43	52	52	52	34
500030MM1020	32	49	38	54	45	76	46	32	36	61
500030MM1030	56	59	41	46	31	37	30	34	60	51
500030MM1040	46	54	32	86	87	55	54	81	41	23
500030MM1050	26	47	45	49	46	75	52	50	46	49
500030MM1060	66	55	60	39	52	46	38	30	35	21
500030MM1070	26	45	30	31	45	45	52	56	57	31
500030MM1080	44	45	27	30	39	37	43	60	40	18
500030MM1090	29	41	40	43	46	30	34	32	22	33
500030MM1100	34	42	40	20	29	28	41	27	27	27
500030MM1110	42	31	43	36	46	20	19	28	35	49
500030MM1120	25	48	49	49	63	41	43	45	30	49
500030MM1130	53	23	22	31	26	36	43	51	38	30
500030MM1140	40	30	43	48	39	38	24	12	35	30
500030MM1150	40	32	23	38	30	19	27	40	46	36
500030MM1160	40	29	34	28	54	53	50	40	34	32
500030MM1170	34	29	22	33	11	29	30	39	31	16
500030MM1180	27	21	30	40	34	27	30	30	37	37
500030MM1190	25	19	38	26	36	47	38	30	28	33
500030MM1200	35	51	21	36	31	36	38	36	35	27

500030MM1210	28	27	26	24	30	30	24	16	17	32
500030MM1220	43	43	36	25	25	22	18	24	22	23
500030MM1230	31	32	24	36	28	30	16	37	21	15
500030MM1240	21	22	27	25	32	32	49	25	34	28
500030MM1250	21	40	32	24	41	40	37	33	26	35
500030MM1260	32	34	39	30	30	27	16	27	15	26
500030MM1270	19	27	11	17	20	16	14	24	19	25
500030MM1280	23	30	17	25	33	23	20	29	26	40
500030MM1290	13	18	36	24	34	24	37	34	29	27
500030MM1300	42	46	37	34	33	32	32	22	22	23
500030MM1310	23	19	25	26	15	12	14	23	21	17
500030MM1320	20	18	22	13	13	15	23	19	16	24
500030MM1330	34	31	39	24	18	8	30	24	41	43
500030MM1340	40	44	35	20	15	22	15	12	20	22
500030MM1350	19	10	20	9	10	13	8	16	31	17
500030MM1360	28	16	6	13	20	27	30	25	17	24
500030MM1370	24	19	28	29	30	22	38	22	32	34
500030MM1380	20	12	17	17	29	29	35	13	23	21
500030MM1390	28	34	27	13	27	29	22	9	12	11
500030MM1400	20	7	13	13	23	22	29	28	27	24
500030MM1410	38	20	13	21	36	28	17	27	11	33
500030MM1420	16	27	20	12	17	26	27	36	27	17
500030MM1430	21	12	13	21	24	24	14	21	14	15
500030MM1440	22	25	24	23	15	22	21	9	13	19
500030MM1450	10	27	29	10	23	27	27	15	15	16
500030MM1460	18	19	18	18	22	23	16	18	16	18
500030MM1470	18	21	17	19	17	23	24	30	28	23
500030MM1480	25	18	28	27	27	29	22	10	14	22
500030MM1490	14	24	33	17	19	19	15	21	13	4
500030MM1500	20	18	13	14	5	11	9	25	21	24
500030MM1510	25	24	8	13	16	14	7	15	21	11
500030MM1520	7	18	21	22	25	18	24	27	13	18
500030MM1530	30	25	21	20	23	12	21	22	17	9
500030MM1540	13	4	13	17	20	21	15	23	14	19
500030MM1550	26	23	34	21	27	11	21	14	27	23
500030MM1560	19	19	18	16	17	17	17	23	12	19
500030MM1570	26	30	38	26	20	27	19	16	26	10
500030MM1580	25	18	19	28	17	21	23	35	23	28
500030MM1590	23	23	28	36	13	21	15	19	24	21
500030MM1600	20	14	22	17	14	29	18	14	21	14
500030MM1610	11	26	31	25	11	25	19	34	27	27
500030MM1620	20	23	22	26	14	17	11	4	17	21
500030MM1630	32	22	26	24	24	43	24	19	11	26
500030MM1640	23	1	13	13	31	41	43	40	20	27
500030MM1650	23	26	27	34	30	20	18	28	19	29
500030MM1660	39	31	31	25	24	37	27	33	41	23
500030MM1670	24	24	24	21	19	33	34	32	18	29
500030MM1680	39	30	32	32	21	15	1	18	15	16
500030MM1690	21	17	10	31	25	23	29	24	25	46
500030MM1700	22	19	11	10	36	21	18	14	21	34
500030MM1710	27	29	42	39	52	45	27	42	24	39
500030MM1720	23	21	17	22	31	20	37	19	37	32
500030MM1730	29	26	35	41	42	33	10	35	19	28
500030MM1740	29	34	37	39	43	38	51	34	21	31
500030MM1750	32	33	28	49	49	48	36	22	33	44
500030MM1760	43	23	37	28	-9999					

500987MM904	83	75	78	75	89	102				
500987MM910	81	83	70	92	92	91	85	61	52	49
500987MM920	63	72	82	54	63	57	71	84	62	27
500987MM930	28	73	73	71	75	50	48	71	108	70
500987MM940	42	51	89	76	96	146	92	48	59	100
500987MM950	29	29	38	62	93	103	72	59	60	103
500987MM960	83	95	66	51	47	47	17	43	45	65
500987MM970	54	61	71	60	67	80	26	58	52	49
500987MM980	36	40	38	31	31	18	38	64	61	65
500987MM990	68	60	66	89	104	119	85	144	76	111
500987MM1000	111	99	72	40	70	63	75	71	93	76
500987MM1010	45	62	80	86	94	63	54	62	43	41
500987MM1020	59	85	53	44	32	59	56	46	61	84
500987MM1030	61	77	58	63	51	106	91	78	137	107
500987MM1040	88	91	55	99	100	69	74	107	91	53
500987MM1050	76	90	89	82	58	83	58	33	50	57
500987MM1060	66	61	61	48	38	37	26	28	40	43
500987MM1070	46	51	34	39	58	60	63	65	57	44
500987MM1080	57	57	40	48	65	42	50	81	58	14
500987MM1090	42	57	76	87	80	51	58	68	38	68
500987MM1100	62	66	50	52	63	34	50	40	36	37
500987MM1110	53	41	61	45	44	34	19	25	26	46
500987MM1120	28	36	70	68	59	52	58	54	27	31
500987MM1130	38	38	34	47	35	39	46	36	39	29
500987MM1140	44	41	45	41	42	41	40	24	41	30
500987MM1150	44	19	16	32	18	18	24	30	46	31
500987MM1160	21	20	35	31	61	52	53	42	43	40
500987MM1170	63	40	37	53	27	45	41	60	47	26
500987MM1180	40	40	43	43	32	25	21	24	27	31
500987MM1190	24	25	41	26	41	53	50	38	45	48
500987MM1200	54	72	31	39	38	44	45	47	44	36
500987MM1210	32	36	45	38	41	46	33	17	14	28
500987MM1220	51	37	38	24	21	20	20	23	16	15
500987MM1230	19	25	19	32	29	22	8	39	14	18
500987MM1240	25	27	33	26	32	24	33	12	15	14
500987MM1250	14	21	22	21	29	27	23	16	12	16
500987MM1260	22	15	30	21	24	18	16	15	20	35
500987MM1270	18	33	17	25	19	8	14	25	24	25
500987MM1280	25	19	10	15	21	16	15	21	23	30
500987MM1290	8	13	19	22	35	33	40	33	28	27
500987MM1300	42	48	35	31	31	37	24	16	18	23
500987MM1310	15	21	25	25	6	6	9	25	20	20
500987MM1320	15	24	24	12	14	8	25	15	16	24
500987MM1330	29	29	29	21	7	8	30	32	28	33
500987MM1340	17	29	20	11	3	16	15	5	23	15
500987MM1350	32	9	25	7	14	9	8	14	24	13
500987MM1360	24	12	4	5	21	29	32	33	13	30
500987MM1370	24	19	21	26	23	16	29	12	31	35
500987MM1380	17	8	10	18	26	20	23	7	26	23
500987MM1390	21	24	25	5	20	30	22	11	21	21
500987MM1400	22	13	11	11	24	17	19	13	17	13
500987MM1410	25	5	6	13	26	14	20	13	8	15
500987MM1420	7	7	8	8	10	7	13	15	5	4
500987MM1430	4	7	9	19	13	16	7	25	4	12
500987MM1440	12	13	12	5	12	7	19	3	9	13
500987MM1450	5	14	12	5	6	9	14	5	7	11

500987MM1460	12	13	9	11	9	13	6	13	14	12
500987MM1470	12	8	9	8	8	12	13	14	13	8
500987MM1480	11	4	5	12	9	5	13	7	8	14
500987MM1490	8	11	12	11	9	6	9	5	9	9
500987MM1500	10	14	11	8	7	14	12	12	13	14
500987MM1510	10	8	7	11	12	14	10	11	7	9
500987MM1520	9	8	10	12	11	5	10	3	7	4
500987MM1530	5	7	7	2	6	8	16	17	14	12
500987MM1540	13	6	4	5	8	2	9	9	6	3
500987MM1550	5	7	7	10	10	10	5	4	8	13
500987MM1560	13	3	15	12	11	11	10	10	12	-9999
500771MM1054	28	52	63	70	66	46				
500771MM1060	72	63	59	56	76	70	69	51	54	37
500771MM1070	52	61	27	26	41	32	35	32	34	41
500771MM1080	42	48	32	31	58	52	69	118	76	28
500771MM1090	48	68	125	67	47	32	40	46	28	43
500771MM1100	32	42	48	37	50	38	46	36	14	32
500771MM1110	53	48	56	42	39	37	17	27	31	40
500771MM1120	31	42	57	44	46	38	42	47	28	42
500771MM1130	39	25	43	61	46	55	46	40	30	28
500771MM1140	55	46	45	51	49	58	44	11	45	35
500771MM1150	46	24	12	34	28	24	33	48	70	77
500771MM1160	52	33	50	31	98	73	63	55	35	45
500771MM1170	60	41	32	44	27	42	35	50	54	16
500771MM1180	45	59	54	59	56	38	44	49	53	37
500771MM1190	38	29	53	32	44	64	67	55	36	43
500771MM1200	44	75	40	45	46	63	54	60	59	45
500771MM1210	48	42	49	35	57	45	30	14	31	52
500771MM1220	86	87	62	41	45	35	28	32	25	25
500771MM1230	29	39	26	42	38	35	21	46	27	28
500771MM1240	22	32	46	40	47	50	58	25	35	33
500771MM1250	20	41	39	34	53	62	68	52	33	45
500771MM1260	57	45	57	50	36	32	31	39	43	59
500771MM1270	43	57	32	49	48	21	26	42	28	30
500771MM1280	31	38	29	26	51	35	35	47	45	66
500771MM1290	23	32	44	25	35	27	39	37	30	29
500771MM1300	53	72	53	44	52	40	44	27	29	27
500771MM1310	40	37	29	20	8	5	6	12	7	9
500771MM1320	12	14	15	13	9	16	32	26	24	15
500771MM1330	25	36	46	26	7	7	23	19	17	23
500771MM1340	14	22	34	19	19	17	6	12	14	18
500771MM1350	22	11	23	20	24	17	3	12	18	8
500771MM1360	22	12	2	15	20	17	20	26	20	28
500771MM1370	21	21	27	33	41	43	37	31	55	69
500771MM1380	40	18	27	26	31	38	34	16	38	36
500771MM1390	35	35	33	12	28	32	28	26	24	33
500771MM1400	36	25	22	21	27	8	31	32	34	43
500771MM1410	51	29	21	19	53	44	31	26	18	27
500771MM1420	12	33	21	18	32	38	48	43	31	23
500771MM1430	28	22	5	16	17	26	19	27	18	18
500771MM1440	21	30	31	23	27	31	34	17	22	16
500771MM1450	15	21	23	14	20	32	44	31	19	25
500771MM1460	33	37	36	19	21	23	27	20	19	20
500771MM1470	37	38	42	35	40	43	46	61	39	48
500771MM1480	57	34	34	40	44	49	36	15	15	30
500771MM1490	20	35	54	40	35	47	32	39	37	8

500771MM1500	21	25	29	28	13	21	27	43	30	34
500771MM1510	41	41	33	42	30	32	18	28	39	21
500771MM1520	12	29	26	34	24	19	29	25	13	19
500771MM1530	29	27	15	25	31	19	31	38	34	31
500771MM1540	23	22	30	53	53	43	32	29	30	37
500771MM1550	39	41	38	30	51	34	43	34	50	34
500771MM1560	32	32	21	28	28	24	11	19	6	19
500771MM1570	28	46	57	50	39	58	44	47	64	38
500771MM1580	57	54	39	44	34	39	36	42	34	43
500771MM1590	35	44	42	52	31	38	31	41	46	51
500771MM1600	55	31	49	39	39	76	55	39	48	28
500771MM1610	22	59	58	70	40	49	51	72	82	83
500771MM1620	75	74	31	48	31	34	24	14	22	24
500771MM1630	48	33	37	40	32	51	36	33	15	42
500771MM1640	20	8	10	8	28	40	50	53	32	46
500771MM1650	31	42	37	49	45	20	13	34	34	34
500771MM1660	46	39	43	55	24	29	21	35	26	27
500771MM1670	35	33	28	16	18	38	31	38	19	28
500771MM1680	42	36	34	27	29	20	3	21	19	20
500771MM1690	24	28	15	30	30	19	29	31	34	60
500771MM1700	3	17	17	19	37	29	33	14	35	43
500771MM1710	30	27	36	34	32	36	29	46	29	27
500771MM1720	18	11	14	13	24	6	20	8	30	16
500771MM1730	18	27	30	52	60	41	48	75	33	45
500771MM1740	47	35	47	46	48	55	84	88	50	42
500771MM1750	13	5	3	18	27	42	53	48	58	69
500771MM1760	58	30	41	21	21	-9999				
500790MM1249	14									
500790MM1250	14	17	12	9	16	14	13	13	7	12
500790MM1260	19	13	16	13	16	16	14	18	18	26
500790MM1270	17	29	18	24	20	13	16	21	22	29
500790MM1280	25	27	11	23	24	23	16	22	22	27
500790MM1290	8	9	18	18	19	23	25	14	13	17
500790MM1300	17	15	11	13	10	10	12	7	11	15
500790MM1310	13	19	20	19	8	11	13	18	17	11
500790MM1320	12	13	9	8	8	9	17	11	12	10
500790MM1330	19	22	16	14	5	6	25	18	16	19
500790MM1340	12	22	15	7	3	9	11	4	11	10
500790MM1350	14	3	12	2	8	8	4	9	14	11
500790MM1360	12	2	5	7	14	18	22	31	16	20
500790MM1370	17	13	22	19	14	10	11	9	15	12
500790MM1380	8	5	4	2	11	12	11	7	18	12
500790MM1390	14	18	17	4	11	13	8	10	13	13
500790MM1400	6	5	12	5	12	14	13	9	11	7
500790MM1410	5	5	-9999							
500123MM1047	43	67	67							
500123MM1050	53	90	74	70	84	70	75	64	81	83
500123MM1060	108	66	87	63	78	86	82	55	82	83
500123MM1070	57	67	46	51	62	51	62	69	68	55
500123MM1080	69	64	49	44	53	43	41	69	59	28
500123MM1090	60	60	76	57	56	36	64	70	42	70
500123MM1100	57	64	68	57	64	56	63	47	47	55
500123MM1110	74	66	69	52	65	48	47	34	37	45
500123MM1120	31	40	51	61	68	68	67	50	29	38
500123MM1130	36	34	39	56	54	59	54	46	52	42
500123MM1140	74	61	57	61	61	81	68	59	91	67

500123MM1150	71	64	52	73	36	34	50	63	74	64
500123MM1160	66	48	57	50	101	95	78	66	50	48
500123MM1170	57	40	34	45	31	50	44	66	68	40
500123MM1180	66	71	68	69	49	47	35	43	45	45
500123MM1190	34	44	57	27	45	75	72	43	38	56
500123MM1200	50	60	31	40	44	58	47	42	38	39
500123MM1210	37	37	49	29	40	41	34	22	31	42
500123MM1220	58	50	62	42	49	52	45	54	45	45
500123MM1230	47	57	38	43	40	47	28	54	26	32
500123MM1240	40	47	59	63	71	71	90	38	43	42
500123MM1250	33	58	52	49	75	84	73	64	40	50
500123MM1260	55	54	68	50	52	34	31	39	35	69
500123MM1270	56	74	54	62	65	31	35	54	42	38
500123MM1280	38	48	27	37	55	49	50	57	51	88
500123MM1290	33	34	54	40	53	42	70	57	56	55
500123MM1300	69	67	43	56	55	54	67	53	48	61
500123MM1310	53	57	59	59	25	34	27	51	47	35
500123MM1320	46	49	64	34	32	31	47	34	35	43
500123MM1330	78	64	77	71	32	28	69	47	47	53
500123MM1340	38	56	50	28	25	39	38	21	38	42
500123MM1350	44	21	38	11	19	17	11	15	29	19
500123MM1360	42	19	8	16	23	34	36	46	30	51
500123MM1370	40	33	50	52	47	41	56	28	44	54
500123MM1380	33	15	16	15	35	48	46	23	54	39
500123MM1390	47	53	51	14	26	36	31	14	29	36
500123MM1400	36	16	10	7	38	23	30	37	40	27
500123MM1410	39	14	11	33	58	42	52	50	40	64
500123MM1420	39	46	38	40	28	36	51	47	43	22
500123MM1430	22	29	25	37	38	47	31	58	26	-9999
500110MM1143	123	130	130	85	46	75	78			
500110MM1150	97	61	54	101	42	56	63	90	133	104
500110MM1160	113	84	73	67	169	128	116	104	36	86
500110MM1170	110	80	76	113	76	98	94	126	129	72
500110MM1180	124	107	113	151	103	72	54	46	49	92
500110MM1190	70	38	44	44	60	95	97	71	73	80
500110MM1200	92	129	71	87	91	92	92	80	94	80
500110MM1210	47	47	50	26	45	54	35	27	37	57
500110MM1220	94	88	74	41	69	58	45	65	43	48
500110MM1230	41	50	48	61	42	52	24	51	17	31
500110MM1240	24	31	32	41	59	61	98	53	57	49
500110MM1250	25	57	49	29	61	73	68	63	43	54
500110MM1260	77	56	79	68	44	29	25	30	26	38
500110MM1270	28	68	40	42	37	17	30	45	46	45
500110MM1280	44	53	46	46	52	40	21	32	42	98
500110MM1290	33	54	63	44	58	56	68	60	35	24
500110MM1300	56	119	106	81	64	46	59	42	28	23
500110MM1310	11	25	37	27	1	3	6	19	20	33
500110MM1320	40	33	40	25	27	28	38	33	25	29
500110MM1330	56	81	104	76	50	45	88	71	69	68
500110MM1340	44	77	84	52	16	66	43	20	52	39
500110MM1350	41	32	47	16	29	30	21	18	37	34
500110MM1360	51	45	19	31	27	47	45	56	45	63
500110MM1370	54	55	73	67	82	77	78	31	57	76
500110MM1380	45	14	25	21	70	77	86	46	66	61
500110MM1390	62	82	63	25	40	7	29	28	42	46
500110MM1400	66	32	21	17	25	10	25	23	37	29

500110MM1410	45	10	5	27	50	58	54	58	32	72
500110MM1420	44	50	28	14	23	24	51	54	41	49
500110MM1430	26	33	30	46	53	47	34	55	19	23
500110MM1440	40	55	68	64	62	81	83	9	57	42
500110MM1450	36	51	53	16	33	47	84	60	56	54
500110MM1460	69	52	61	10	30	42	51	42	44	48
500110MM1470	53	48	45	30	38	53	61	61	49	50
500110MM1480	60	40	49	73	84	84	58	26	8	44
500110MM1490	32	41	55	52	33	37	43	46	43	2
500110MM1500	36	38	38	45	12	25	21	25	44	41
500110MM1510	46	74	43	70	63	58	21	53	45	25
500110MM1520	19	35	42	43	19	22	39	45	9	34
500110MM1530	65	49	50	53	62	25	53	54	46	31
500110MM1540	26	27	29	37	45	39	41	27	28	30
500110MM1550	45	48	46	49	39	30	48	29	50	43
500110MM1560	42	45	41	20	30	18	8	18	8	18
500110MM1570	35	42	51	35	29	31	31	31	5	9
500110MM1580	35	23	32	36	32	33	31	32	27	31
500110MM1590	28	29	37	39	34	30	29	32	31	42
500110MM1600	45	36	44	25	35	34	19	10	31	19
500110MM1610	1	29	29	35	9	34	26	47	45	22
500110MM1620	38	49	17	24	13	27	18	15	28	39
500110MM1630	42	47	55	37	61	21	14	23	21	43
500110MM1640	-9999									
500108MM989	62									
500108MM990	103	97	89	83	131	98	75	113	82	87
500108MM1000	101	105	84	98	97	93	83	72	102	73
500108MM1010	36	54	80	79	66	35	37	36	29	29
500108MM1020	41	87	54	72	50	74	50	21	22	38
500108MM1030	36	50	32	28	28	60	68	51	82	59
500108MM1040	53	57	35	66	64	34	32	55	49	22
500108MM1050	27	46	45	41	39	57	43	22	35	49
500108MM1060	59	42	39	35	34	40	42	33	29	33
500108MM1070	36	43	29	35	52	50	45	41	40	28
500108MM1080	41	44	32	29	47	25	40	58	37	15
500108MM1090	37	35	68	58	49	29	40	41	23	40
500108MM1100	21	29	23	26	34	30	26	25	25	33
500108MM1110	32	34	37	31	39	33	20	30	27	29
500108MM1120	23	23	30	45	51	51	53	47	34	42
500108MM1130	41	37	42	41	42	54	43	36	29	33
500108MM1140	51	37	36	40	40	41	34	11	34	24
500108MM1150	34	15	18	31	19	25	35	37	42	33
500108MM1160	24	17	26	16	44	42	44	34	32	29
500108MM1170	38	25	24	34	17	28	21	32	36	16
500108MM1180	37	32	42	40	30	26	23	26	31	23
500108MM1190	20	24	36	22	32	49	41	36	27	42
500108MM1200	45	68	36	55	41	48	42	36	39	37
500108MM1210	32	38	42	23	31	27	18	13	23	28
500108MM1220	44	32	33	22	25	23	26	27	21	31
500108MM1230	33	39	26	35	23	24	18	37	17	24
500108MM1240	21	22	33	28	31	28	31	10	20	18
500108MM1250	27	51	43	33	46	47	42	39	34	41
500108MM1260	42	30	45	36	41	31	19	30	29	44
500108MM1270	27	43	30	35	36	16	17	31	19	19
500108MM1280	20	33	14	13	36	26	26	33	27	38
500108MM1290	11	21	36	29	45	35	50	49	42	43

500108MM1300	65	79	54	57	49	45	48	36	33	39
500108MM1310	37	39	38	46	21	26	25	36	24	27
500108MM1320	33	45	49	32	25	31	43	36	14	25
500108MM1330	50	40	50	50	33	27	71	60	61	60
500108MM1340	40	57	52	37	27	46	35	31	44	40
500108MM1350	45	24	43	30	34	39	21	29	44	29
500108MM1360	44	34	13	34	52	50	44	54	41	59
500108MM1370	51	40	53	52	53	53	57	35	51	53
500108MM1380	38	16	27	29	48	51	48	29	53	35
500108MM1390	44	51	41	19	36	43	33	14	26	28
500108MM1400	24	12	15	20	24	18	24	31	34	26
500108MM1410	35	12	15	26	38	40	37	36	26	56
500108MM1420	29	33	24	22	29	26	44	43	32	29
500108MM1430	19	26	20	31	36	37	26	39	27	30
500108MM1440	46	40	37	37	30	36	36	15	31	28
500108MM1450	18	34	38	27	41	43	46	28	27	29
500108MM1460	40	39	44	39	43	44	36	44	36	38
500108MM1470	43	32	47	38	44	58	56	66	46	47
500108MM1480	48	32	47	69	64	78	81	36	28	41
500108MM1490	34	45	51	34	34	36	33	48	43	15
500108MM1500	44	56	48	51	20	20	27	28	43	35
500108MM1510	39	58	33	60	60	44	23	36	53	36
500108MM1520	22	38	44	39	30	24	29	30	10	28
500108MM1530	46	55	46	53	53	29	50	59	57	38
500108MM1540	37	42	40	41	49	52	30	30	33	54
500108MM1550	48	58	59	37	40	32	38	30	49	31
500108MM1560	43	27	28	19	18	28	29	32	16	30
500108MM1570	44	38	49	38	35	52	40	52	68	56
500108MM1580	55	48	54	38	25	32	25	24	23	21
500108MM1590	20	32	35	37	18	34	19	30	45	31
500108MM1600	34	37	32	34	21	34	24	14	25	19
500108MM1610	26	35	35	26	11	34	9	26	55	33
500108MM1620	47	50	21	45	23	26	20	9	31	20
500108MM1630	38	30	32	30	38	42	26	18	13	32
500108MM1640	14	4	1	9	33	49	53	60	27	48
500108MM1650	25	29	27	42	39	19	24	28	21	-9999
500231MM1278	122	123								
500231MM1280	113	205	141	174	136	121	165	202	197	124
500231MM1290	75	121	90	92	97	113	145	141	132	118
500231MM1300	92	108	115	83	96	78	68	58	75	66
500231MM1310	80	83	89	89	59	72	49	82	74	67
500231MM1320	61	76	73	48	58	77	93	73	62	96
500231MM1330	119	98	98	92	65	52	87	88	76	99
500231MM1340	77	87	70	60	41	57	46	55	56	45
500231MM1350	56	37	54	39	50	47	28	49	44	38
500231MM1360	48	65	30	34	49	60	58	65	54	47
500231MM1370	43	52	44	61	54	51	42	48	60	42
500231MM1380	37	35	28	25	37	45	54	29	32	50
500231MM1390	30	24	37	23	33	40	33	20	27	27
500231MM1400	32	8	15	26	31	23	39	33	31	23
500231MM1410	38	20	10	26	20	32	25	22	20	29
500231MM1420	15	21	24	24	22	16	28	30	29	29
500231MM1430	24	34	31	29	28	29	23	33	19	30
500231MM1440	37	39	37	30	-9999					
100000MM1244	27	30	22	8	16	29				
100000MM1250	34	40	42	40	49	64	53	54	40	33

100000MM1260	20	30	15	10	23	14	16	35	23	47
100000MM1270	51	63	53	70	68	54	47	67	58	57
100000MM1280	52	72	62	58	98	69	75	94	114	76
100000MM1290	32	59	55	65	78	63	74	72	86	89
100000MM1300	87	100	84	57	67	75	43	39	57	75
100000MM1310	95	92	98	85	38	70	72	106	96	70
100000MM1320	82	98	120	82	97	91	108	96	76	118
100000MM1330	120	106	108	90	32	55	101	95	100	119
100000MM1340	110	99	80	84	56	66	73	58	64	79
100000MM1350	81	68	81	74	90	85	59	74	63	64
100000MM1360	63	75	65	39	73	94	89	129	118	119
100000MM1370	101	75	94	81	68	99	100	60	91	66
100000MM1380	71	63	57	45	36	48	56	29	61	72
100000MM1390	55	58	35	36	36	56	62	55	58	25
100000MM1400	42	25	33	33	43	31	35	34	38	44
100000MM1410	52	34	36	48	62	61	52	53	15	47
100000MM1420	42	41	46	12	25	19	35	43	30	29
100000MM1430	36	56	38	54	-9999					
500060MM1309	94									
500060MM1310	160	122	101	101	115	74	128	128	88	73
500060MM1320	108	142	77	88	110	120	171	162	86	150
500060MM1330	210	155	137	137	102	120	136	99	83	85
500060MM1340	107	103	98	81	80	109	97	104	98	87
500060MM1350	103	69	94	55	85	74	65	69	54	70
500060MM1360	68	49	50	18	70	83	89	105	70	66
500060MM1370	60	43	69	75	68	78	100	98	91	78
500060MM1380	73	52	42	50	55	60	51	26	42	55
500060MM1390	54	61	43	26	41	62	69	40	42	44
500060MM1400	38	33	41	60	55	47	27	44	50	41
500060MM1410	28	24	24	30	36	42	39	43	5	35
500060MM1420	36	34	26	19	32	42	59	57	54	34
500060MM1430	38	40	31	39	49	42	35	27	8	17
500060MM1440	30	39	50	46	50	39	42	30	32	34
500060MM1450	20	39	32	40	55	61	58	52	33	43
500060MM1460	56	58	59	42	47	66	51	30	30	30
500060MM1470	57	55	48	46	55	46	40	34	41	47
500060MM1480	47	38	42	7	20	18	38	13	8	48
500060MM1490	51	43	40	40	42	39	58	48	62	33
500060MM1500	45	51	63	46	19	41	40	56	46	51
500060MM1510	68	80	69	46	70	43	54	59	53	22
500060MM1520	25	34	26	25	33	28	19	26	4	17
500060MM1530	16	30	25	11	19	27	62	53	41	22
500060MM1540	23	7	8	11	25	34	28	16	25	18
500060MM1550	20	18	13	20	10	10	17	15	15	19
500060MM1560	13	11	8	8	6	5	4	4	9	8
500060MM1570	12	15	8	16	20	14	16	10	12	8
500060MM1580	11	9	8	-9999						
500783MM1184	120	77	56	71	78	62				
500783MM1190	48	61	77	48	69	85	75	55	72	65
500783MM1200	74	91	55	71	64	76	57	51	41	49
500783MM1210	42	48	57	44	48	48	51	30	34	48
500783MM1220	63	68	69	54	53	51	58	56	44	47
500783MM1230	51	51	35	50	48	38	40	64	40	33
500783MM1240	34	34	44	48	66	60	59	33	48	42
500783MM1250	36	58	46	49	60	48	45	41	35	55
500783MM1260	60	60	60	57	60	48	34	56	48	71

500783MM1270	51	67	44	43	45	30	33	57	36	39
500783MM1280	38	42	26	33	58	53	54	74	55	72
500783MM1290	27	25	31	35	41	34	43	39	43	46
500783MM1300	64	57	39	44	39	39	28	20	22	34
500783MM1310	28	38	33	38	25	25	30	49	43	40
500783MM1320	50	54	54	39	35	35	50	42	31	43
500783MM1330	75	72	78	81	37	35	62	48	54	67
500783MM1340	51	61	48	31	41	54	50	46	44	53
500783MM1350	52	27	46	35	38	51	33	59	72	49
500783MM1360	77	40	39	45	63	66	60	59	41	52
500783MM1370	48	28	43	54	49	35	48	29	42	40
500783MM1380	28	18	22	13	15	26	25	13	37	28
500783MM1390	32	55	41	22	33	48	35	25	34	28
500783MM1400	34	20	23	27	42	38	35	27	28	38
500783MM1410	36	7	15	18	34	27	24	28	18	34
500783MM1420	10	21	17	19	23	39	43	34	43	24
500783MM1430	30	29	27	27	30	27	22	31	30	40
500783MM1440	28	30	46	49	39	37	38	33	23	45
500783MM1450	27	33	37	24	44	39	39	23	21	22
500783MM1460	22	14	14	20	26	30	26	30	21	30
500783MM1470	39	36	33	29	23	31	25	41	34	25
500783MM1480	22	7	21	18	21	21	21	5	17	26
500783MM1490	29	47	50	33	36	46	61	50	40	22
500783MM1500	32	40	45	45	31	44	33	58	51	34
500783MM1510	40	49	42	54	41	36	15	26	34	16
500783MM1520	22	30	34	36	33	26	28	22	23	33
500783MM1530	39	26	30	24	32	22	38	44	27	22
500783MM1540	30	35	41	44	42	34	25	21	34	38
500783MM1550	39	31	22	24	28	19	35	27	40	22
500783MM1560	21	21	12	6	8	5	4	20	31	31
500783MM1570	45	49	53	29	26	44	26	30	39	22
500783MM1580	29	16	24	33	22	36	40	42	25	26
500783MM1590	26	31	26	29	15	20	12	19	34	33
500783MM1600	28	16	24	14	15	23	21	18	24	10
500783MM1610	28	45	51	34	22	24	26	45	38	42
500783MM1620	32	35	25	28	14	21	14	15	23	12
500783MM1630	34	30	22	15	23	39	24	17	19	45
500783MM1640	9	4	6	8	26	19	35	35	12	22
500783MM1650	22	26	24	39	36	19	13	33	17	38
500783MM1660	40	23	22	33	20	34	28	38	28	23
500783MM1670	23	20	14	17	22	30	22	16	13	16
500783MM1680	25	28	25	30	19	23	13	21	13	20
500783MM1690	27	22	25	35	27	22	24	26	20	44
500783MM1700	23	19	14	15	34	19	22	9	32	31
500783MM1710	30	29	38	35	36	36	29	38	33	38
500783MM1720	32	39	40	23	52	38	54	43	53	47
500783MM1730	47	47	48	62	54	47	51	55	52	51
500783MM1740	36	37	54	42	43	48	55	52	40	59
500783MM1750	39	15	11	30	43	29	22	21	30	20
500783MM1760	16	17	17	-9999						
500911MM1283	31	43	77	66	68	60	91			
500911MM1290	38	37	50	41	79	54	86	76	66	52
500911MM1300	72	95	58	57	58	43	44	27	19	21
500911MM1310	33	37	46	53	14	17	47	58	18	18
500911MM1320	22	5	25	18	33	16	45	42	44	40
500911MM1330	46	50	45	41	11	7	30	41	31	36

500911MM1340	22	58	51	26	15	41	47	23	52	36
500911MM1350	46	17	29	10	24	25	7	16	86	34
500911MM1360	37	30	7	40	47	54	53	71	57	87
500911MM1370	92	72	92	96	97	73	92	40	73	83
500911MM1380	30	6	11	31	62	55	56	21	43	40
500911MM1390	44	48	38	5	20	35	31	6	2	22
500911MM1400	25	6	2	27	15	24	17	3	34	6
500911MM1410	22	13	4	19	36	31	23	23	6	42
500911MM1420	10	2	19	15	3	17	13	33	39	5
500911MM1430	12	3	16	12	2	31	35	37	11	49
500911MM1440	28	34	29	17	29	22	15	42	4	48
500911MM1450	30	27	68	70	16	42	43	46	9	10
500911MM1460	20	31	31	20	30	16	15	23	33	28
500911MM1470	30	45	57	45	44	51	68	63	70	54
500911MM1480	70	52	34	33	72	61	78	57	18	17
500911MM1490	41	59	27	65	49	39	36	50	45	40
500911MM1500	15	34	22	32	44	15	34	32	49	48
500911MM1510	41	49	66	23	71	77	45	11	53	53
500911MM1520	19	3	25	37	36	28	18	36	40	19
500911MM1530	40	50	52	42	40	56	19	53	46	24
500911MM1540	12	22	7	21	24	27	22	15	10	13
500911MM1550	23	32	22	28	23	25	14	24	2	24
500911MM1560	18	2	9	17	9	11	19	16	14	6
500911MM1570	16	21	24	2	25	21	37	25	24	36
500911MM1580	17	34	19	28	34	20	29	49	56	34
500911MM1590	53	48	52	45	54	16	19	17	33	43
500911MM1600	26	35	21	37	17	15	23	8	11	23
500911MM1610	5	25	-9999							
500360MM993	106	171	97	101	137	113	156			
500360MM1000	123	154	139	135	193	203	149	115	154	119
500360MM1010	118	133	146	125	134	78	91	105	68	55
500360MM1020	53	77	50	53	60	82	73	53	60	75
500360MM1030	61	85	57	56	83	93	76	67	115	90
500360MM1040	68	90	52	92	82	47	55	83	76	50
500360MM1050	71	77	81	62	90	93	69	66	63	83
500360MM1060	106	72	86	56	50	53	67	52	58	50
500360MM1070	56	55	54	47	75	61	51	66	68	51
500360MM1080	58	53	29	40	54	22	40	61	47	8
500360MM1090	44	50	86	62	68	37	75	76	62	58
500360MM1100	61	57	46	37	48	30	46	41	31	68
500360MM1110	67	46	62	42	39	25	16	27	33	34
500360MM1120	20	48	71	69	37	48	40	35	17	11
500360MM1130	34	18	31	37	33	31	41	24	19	38
500360MM1140	66	53	54	51	38	43	37	3	16	28
500360MM1150	44	34	30	43	21	4	31	56	75	60
500360MM1160	33	14	22	19	39	32	29	16	13	23
500360MM1170	28	9	8	27	15	23	15	20	18	1
500360MM1180	20	21	23	29	13	11	8	14	20	25
500360MM1190	16	10	26	9	23	36	30	6	11	26
500360MM1200	23	47	19	33	37	39	38	31	31	22
500360MM1210	18	26	38	19	24	25	16	5	5	28
500360MM1220	38	35	34	25	26	19	12	20	4	23
500360MM1230	24	28	21	33	25	21	2	40	14	15
500360MM1240	23	23	36	34	13	19	33	22	25	17
500360MM1250	11	32	38	29	44	35	27	15	7	13
500360MM1260	17	5	19	15	24	18	8	18	12	19

500360MM1270	9	19	14	4	2	5	12	21	11	8
500360MM1280	13	18	16	26	34	28	30	36	33	43
500360MM1290	9	12	29	23	29	24	36	38	28	19
500360MM1300	48	41	43	37	50	39	36	21	14	27
500360MM1310	30	31	26	19	5	8	6	25	20	9
500360MM1320	10	19	24	14	11	13	24	19	12	21
500360MM1330	36	18	24	21	5	4	34	26	29	38
500360MM1340	15	24	23	18	10	16	14	10	30	30
500360MM1350	37	22	44	26	26	30	18	35	53	32
500360MM1360	45	28	20	32	40	25	30	29	32	44
500360MM1370	36	42	47	47	36	23	33	25	40	42
500360MM1380	24	10	7	11	36	35	32	16	31	24
500360MM1390	27	32	29	14	39	32	34	9	7	14
500360MM1400	11	4	5	12	16	6	2	18	29	20
500360MM1410	37	16	12	27	32	32	30	23	9	41
500360MM1420	16	23	18	16	17	20	29	39	25	22
500360MM1430	20	17	25	35	37	38	27	45	21	31
500360MM1440	31	28	27	21	15	15	23	15	20	29
500360MM1450	16	38	46	9	31	27	29	14	26	21
500360MM1460	34	23	30	35	37	25	21	27	26	26
500360MM1470	28	36	22	23	22	35	41	39	37	42
500360MM1480	48	24	25	37	33	28	32	12	29	24
500360MM1490	28	45	39	32	41	58	26	33	23	1
500360MM1500	29	29	30	24	6	42	39	43	59	36
500360MM1510	39	48	25	44	34	29	17	36	37	24
500360MM1520	25	35	44	40	29	22	34	38	14	28
500360MM1530	43	52	59	60	61	26	39	50	32	18
500360MM1540	15	13	19	36	32	42	44	39	43	28
500360MM1550	43	33	38	35	47	25	31	9	28	26
500360MM1560	22	33	19	19	25	23	24	35	24	32
500360MM1570	39	57	74	59	39	44	25	29	29	13
500360MM1580	28	18	26	33	27	44	53	58	45	49
500360MM1590	48	57	49	49	27	24	18	27	38	31
500360MM1600	35	23	38	29	20	52	29	23	27	25
500360MM1610	17	32	37	35	1	30	23	37	52	44
500360MM1620	49	56	47	39	29	34	12	20	42	40
500360MM1630	63	38	35	38	42	59	48	37	10	49
500360MM1640	41	28	9	8	25	37	-9999			
500762MM847	125	129	149							
500762MM850	127	128	78	73	122	104	127	112	75	103
500762MM860	116	70	130	128	132	96	83	71	90	94
500762MM870	100	106	115	84	106	110	80	81	99	88
500762MM880	88	73	74	39	37	34	42	60	32	47
500762MM890	60	79	51	74	88	65	91	94	146	103
500762MM900	61	98	69	48	35	49	46	64	58	72
500762MM910	32	43	65	85	67	78	75	66	47	47
500762MM920	57	58	68	70	82	39	61	63	34	21
500762MM930	28	68	54	57	63	106	69	84	135	107
500762MM940	108	69	76	59	50	90	40	33	52	81
500762MM950	24	48	63	55	63	64	58	50	48	80
500762MM960	53	57	46	31	42	50	33	37	42	34
500762MM970	39	40	35	38	46	41	22	66	70	72
500762MM980	68	59	51	38	39	16	29	44	48	40
500762MM990	62	68	65	65	97	102	74	150	91	96
500762MM1000	102	97	82	73	95	69	72	101	111	87
500762MM1010	52	52	67	68	58	42	32	38	31	35

500762MM1020	48	57	58	81	53	67	84	71	78	73
500762MM1030	44	43	34	49	28	48	55	44	77	45
500762MM1040	37	59	21	83	92	52	64	110	92	53
500762MM1050	51	62	69	67	55	92	62	26	62	77
500762MM1060	98	90	66	67	81	80	78	56	52	55
500762MM1070	52	75	51	40	75	80	88	82	65	56
500762MM1080	90	75	58	52	69	47	64	65	42	16
500762MM1090	46	41	54	54	39	20	29	32	19	34
500762MM1100	21	30	27	18	40	24	28	31	27	34
500762MM1110	46	33	46	32	50	42	31	37	35	56
500762MM1120	37	33	81	79	87	77	81	71	49	60
500762MM1130	45	43	44	44	43	53	48	51	35	32
500762MM1140	46	37	45	36	37	38	45	8	46	43
500762MM1150	54	31	37	49	22	20	46	53	65	41
500762MM1160	39	22	38	32	63	71	54	48	44	32
500762MM1170	47	37	31	32	20	39	37	56	60	40
500762MM1180	51	42	44	47	30	27	30	32	37	43
500762MM1190	28	25	39	21	37	63	78	58	75	66
500762MM1200	70	81	37	42	42	47	37	49	48	40
500762MM1210	41	48	60	43	45	46	35	15	38	56
500762MM1220	69	64	48	47	49	45	43	68	40	44
500762MM1230	54	51	32	35	30	30	20	48	25	22
500762MM1240	26	34	44	39	47	53	49	24	32	37
500762MM1250	40	51	45	43	57	62	59	57	51	55
500762MM1260	72	55	78	59	60	44	49	39	40	70
500762MM1270	39	56	27	46	28	19	19	46	36	46
500762MM1280	37	44	54	49	82	46	34	46	50	66
500762MM1290	15	38	48	33	44	41	73	83	68	60
500762MM1300	74	77	66	51	60	62	51	40	53	43
500762MM1310	36	35	44	30	19	20	25	38	31	36
500762MM1320	28	45	43	28	28	36	49	42	49	62
500762MM1330	95	70	78	60	27	28	72	54	47	66
500762MM1340	35	44	46	32	30	61	54	55	67	43
500762MM1350	65	37	45	31	37	27	29	34	66	21
500762MM1360	36	35	18	38	45	58	70	97	70	87
500762MM1370	68	54	59	63	53	49	52	43	81	66
500762MM1380	44	24	17	28	57	54	45	22	36	37
500762MM1390	40	42	35	16	37	34	19	10	31	26
500762MM1400	25	12	16	20	33	26	37	29	38	42
500762MM1410	73	27	15	38	48	43	35	25	20	44
500762MM1420	24	19	17	17	24	42	64	44	47	32
500762MM1430	20	28	32	38	45	41	25	44	13	25
500762MM1440	41	47	47	56	45	49	54	10	29	25
500762MM1450	32	34	24	18	35	46	69	54	46	48
500762MM1460	63	49	32	32	26	30	32	28	30	34
500762MM1470	44	29	34	22	36	42	40	45	51	45
500762MM1480	43	31	44	44	38	41	49	29	25	52
500762MM1490	35	49	54	54	41	38	31	33	38	12
500762MM1500	12	29	23	30	10	30	21	42	39	43
500762MM1510	48	49	22	51	59	38	32	60	71	38
500762MM1520	23	49	53	39	34	25	40	46	16	33
500762MM1530	56	56	26	34	30	18	28	37	28	22
500762MM1540	21	21	28	35	36	28	33	23	15	22
500762MM1550	24	17	19	18	22	14	17	22	45	31
500762MM1560	21	19	19	11	15	13	19	11	13	10
500762MM1570	18	21	43	38	23	25	15	31	27	20

500762MM1580	31	21	21	23	11	17	30	51	38	27
500762MM1590	18	23	31	31	9	19	22	19	26	33
500762MM1600	29	16	14	17	14	9	9	13	14	4
500762MM1610	4	25	32	27	11	9	15	25	26	23
500762MM1620	26	23	15	20	16	19	13	9	14	12
500762MM1630	21	17	13	13	15	23	15	16	12	24
500762MM1640	13	9	6	6	18	17	29	30	14	33
500762MM1650	19	20	13	17	10	9	8	19	9	20
500762MM1660	35	12	13	32	15	15	19	34	20	16
500762MM1670	12	19	20	18	19	36	34	-9999		
500082MM1263	37	61	49	45	48	26	38			
500082MM1270	17	38	21	41	44	26	31	36	26	29
500082MM1280	32	39	35	39	60	42	86	80	68	77
500082MM1290	26	46	49	36	83	72	104	88	68	63
500082MM1300	66	65	50	47	38	30	25	14	25	29
500082MM1310	48	34	22	29	21	9	10	23	24	16
500082MM1320	25	34	36	19	16	12	32	20	6	22
500082MM1330	47	44	75	77	50	40	80	69	79	79
500082MM1340	57	80	55	43	40	35	36	34	38	37
500082MM1350	31	33	54	42	50	58	35	70	86	60
500082MM1360	73	54	23	39	11	31	34	43	30	36
500082MM1370	45	36	46	42	41	41	35	23	36	29
500082MM1380	26	19	14	10	30	26	31	24	36	41
500082MM1390	50	58	48	30	36	49	38	19	23	25
500082MM1400	24	6	20	24	31	27	24	23	31	29
500082MM1410	41	24	18	23	37	39	26	27	16	46
500082MM1420	25	15	10	14	10	27	33	28	38	34
500082MM1430	25	19	16	31	30	28	19	26	17	22
500082MM1440	19	20	23	32	25	29	19	16	26	29
500082MM1450	16	35	35	15	23	24	32	28	21	20
500082MM1460	24	17	30	29	36	24	24	25	25	20
500082MM1470	23	44	42	30	31	38	38	40	34	57
500082MM1480	70	30	55	61	69	58	47	22	18	52
500082MM1490	59	58	70	78	52	51	52	49	54	11
500082MM1500	38	35	32	36	20	35	31	60	45	43
500082MM1510	25	36	17	31	19	18	18	28	23	1
500082MM1520	1	9	6	10	8	1	3	11	1	8
500082MM1530	10	15	10	13	13	3	15	21	9	4
500082MM1540	8	11	13	9	14	16	11	8	13	19
500082MM1550	28	28	25	22	27	10	14	15	23	18
500082MM1560	19	13	11	1	10	16	12	21	12	19
500082MM1570	33	25	36	36	22	30	10	23	33	19
500082MM1580	37	26	34	26	11	26	23	23	22	19
500082MM1590	17	26	32	29	22	26	23	31	32	29
500082MM1600	28	24	38	21	26	33	29	32	28	17
500082MM1610	7	19	36	27	17	39	21	39	33	33
500082MM1620	28	44	24	34	16	18	8	9	3	4
500082MM1630	24	18	17	17	13	21	14	6	9	26
500082MM1640	14	9	14	9	21	17	16	18	9	17
500082MM1650	10	14	19	28	22	14	17	27	11	25
500082MM1660	34	28	16	24	15	29	13	31	17	12
500082MM1670	20	19	21	8	7	20	20	23	21	24
500082MM1680	31	25	23	15	14	15	3	18	9	10
500082MM1690	15	17	16	35	7	8	20	11	10	47
500082MM1700	21	16	9	21	36	12	29	11	28	26
500082MM1710	19	20	19	23	34	24	12	20	17	28

500082MM1720	17	28	23	23	33	4	19	3	25	15
500082MM1730	23	26	34	43	53	33	41	67	32	-9999
500050MM1254	20	19	27	43	36	34				
500050MM1260	58	51	78	86	65	69	53	79	86	126
500050MM1270	197	151	106	114	96	177	67	96	95	167
500050MM1280	106	130	78	122	93	132	138	92	131	119
500050MM1290	81	95	68	57	64	69	139	127	129	129
500050MM1300	131	118	123	117	89	108	80	53	74	83
500050MM1310	125	98	110	95	77	98	62	94	73	76
500050MM1320	66	77	82	85	92	92	94	82	64	84
500050MM1330	104	94	121	77	51	55	78	67	68	71
500050MM1340	56	80	78	86	56	61	63	66	79	71
500050MM1350	76	62	66	60	65	57	32	49	60	59
500050MM1360	60	64	64	27	63	66	75	99	76	68
500050MM1370	63	51	70	76	64	74	46	48	68	35
500050MM1380	45	38	44	65	55	63	83	47	64	57
500050MM1390	29	37	40	23	29	37	39	36	31	21
500050MM1400	37	5	15	30	44	29	33	29	44	31
500050MM1410	38	19	39	51	45	52	42	42	25	38
500050MM1420	25	35	28	28	26	21	25	35	20	21
500050MM1430	24	21	11	24	27	32	25	45	11	30
500050MM1440	31	40	37	28	40	55	55	44	35	51
500050MM1450	32	32	38	42	28	34	-9999			
600610 737	126	119	152							
600610 740	104	122	114	201	162	159	185	213	237	191
600610 750	229	157	182	248	211	141	155	221	178	171
600610 760	126	200	171	189	167	146	145	164	141	190
600610 770	102	125	160	170	176	200	172	152	106	187
600610 780	127	151	156	109	185	97	93	131	100	110
600610 790	96	97	85	130	114	94	81	92	97	114
600610 800	103	39	66	66	99	59	75	62	52	75
600610 810	70	64	73	45	64	59	56	42	65	56
600610 820	39	53	64	75	80	78	64	33	41	43
600610 830	66	75	74	68	63	34	62	39	36	55
600610 840	63	80	41	43	67	100	76	72	55	47
600610 850	50	46	40	42	46	55	51	41	41	39
600610 860	42	35	53	53	45	60	38	56	36	52
600610 870	55	46	38	15	46	102	82	48	54	48
600610 880	57	49	33	45	49	24	31	30	24	22
600610 890	25	42	32	39	21	24	30	18	32	36
600610 900	41	36	34	29	29	37	24	25	35	30
600610 910	17	35	35	47	42	37	35	39	33	21
600610 920	25	15	23	30	32	29	33	44	41	25
600610 930	41	53	40	42	48	46	29	27	41	42
600610 940	58	28	25	23	36	38	25	21	34	43
600610 950	30	32	34	30	37	24	21	11	28	44
600610 960	36	35	23	33	30	35	38	31	34	21
600610 970	32	33	24	14	41	41	25	47	47	32
600610 980	17	17	19	31	34	27	61	49	31	14
600610 990	29	18	24	33	38	45	29	42	39	52
600610 1000	51	36	37	16	38	39	31	14	22	43
600610 1010	23	39	47	50	42	28	22	16	13	13
600610 1020	9	18	23	33	23	22	30	22	38	31
600610 1030	17	18	14	22	2	19	34	27	20	13
600610 1040	14	18	2	15	23	15	24	22	26	16
600610 1050	2	25	35	20	20	27	24	11	8	20

600610	1060	32	31	26	29	5	26	22	32	20	9
600610	1070	22	30	16	18	12	18	24	22	26	10
600610	1080	43	34	33	24	37	17	29	33	29	18
600610	1090	28	18	29	31	23	17	21	28	25	34
600610	1100	27	19	20	33	39	23	37	34	17	23
600610	1110	31	24	14	30	27	31	20	16	28	48
600610	1120	43	50	46	49	25	43	41	23	14	10
600610	1130	30	28	22	34	34	14	24	23	10	10
600610	1140	19	20	18	18	21	17	13	17	28	16
600610	1150	26	25	16	22	16	2	10	8	14	6
600610	1160	4	18	22	19	17	17	14	20	22	9
600610	1170	7	3	15	19	17	23	17	9	9	17
600610	1180	3	17	12	16	16	16	10	2	17	5
600610	1190	15	13	21	18	13	17	10	9	24	18
600610	1200	17	28	16	21	30	36	31	21	26	17
600610	1210	20	13	31	34	30	26	35	13	28	23
600610	1220	22	31	23	10	18	6	7	32	13	16
600610	1230	37	23	20	26	5	26	23	28	10	15
600610	1240	20	39	25	13	39	26	34	13	24	18
600610	1250	22	23	27	42	23	25	13	10	14	25
600610	1260	20	17	20	18	24	10	3	22	19	29
600610	1270	33	30	14	19	28	6	29	11	27	27
600610	1280	13	3	23	17	29	14	27	22	23	19
600610	1290	7	12	9	18	25	5	20	18	27	11
600610	1300	24	21	12	15	10	15	8	7	23	24
600610	1310	20	27	34	28	17	30	23	37	27	40
600610	1320	27	30	18	21	25	26	34	18	22	21
600610	1330	64	75	70	76	37	54	78	70	73	72
600610	1340	80	85	70	67	44	55	62	53	50	60
600610	1350	66	31	61	22	57	41	23	56	46	32
600610	1360	45	31	24	24	38	56	44	55	29	39
600610	1370	40	19	40	35	32	33	35	32	46	32
600610	1380	33	35	28	19	40	33	27	9	36	37
600610	1390	26	28	21	12	29	34	27	28	29	11
600610	1400	32	14	28	30	44	27	44	40	44	26
600610	1410	29	18	16	22	35	24	17	23	9	29
600610	1420	15	25	25	25	26	20	30	31	18	22
600610	1430	22	16	20	23	19	14	20	26	6	23
600610	1440	22	23	19	20	26	14	23	19	23	21
600610	1450	14	15	14	10	18	26	34	55	38	28
600610	1460	28	30	28	21	25	30	17	23	15	22
600610	1470	26	27	21	21	23	30	21	18	13	24
600610	1480	15	10	20	22	17	15	27	15	22	30
600610	1490	35	27	25	32	18	16	29	37	38	18
600610	1500	25	13	33	28	8	20	7	42	26	28
600610	1510	24	36	17	24	39	19	28	27	35	13
600610	1520	11	42	27	26	10	22	24	32	17	17
600610	1530	26	24	24	14	13	14	27	38	20	19
600610	1540	19	15	23	20	34	23	20	12	25	23
600610	1550	42	33	40	17	23	23	26	29	38	19
600610	1560	32	28	16	18	13	6	23	30	13	23
600610	1570	29	27	47	29	14	41	31	37	40	27
600610	1580	44	24	49	50	28	43	46	30	39	16
600610	1590	19	32	29	17	15	22	28	25	22	21
600610	1600	-9999									

Supplementary Information, Table 2|δ^{13}C measurements per mille (‰) versus VPDB on samples from GOR-3E, GOR-3S, GOR-77A, GOR-82A and GOR-87B identified by Gordion (GOR) Relative Year (RY).

	Sample				
	GOR-3-E	GOR-3-S	GOR-77-A	GOR-82-A	GOR-87-B
GOR RY	δ^{13}C ‰ vs. VPDB	δ^{13}C ‰ vs. VPDB	δ^{13}C ‰ vs. VPDB	δ^{13}C ‰ vs. VPDB	δ^{13}C ‰ vs. VPDB
1049		-19.4869753956151			
1050		-19.8953882265429			
1051		-19.9316262954741			
1052		-19.9708025862106			
1053		-19.7553329871600			
1054		-19.7014655873973			
1055		-19.8297679395593			
1056		-19.5653279770880			
1057		-19.8728618593694			
1058		-20.0658050912466			
1059		-20.0599286476361			
1060		-19.9864731025052			
1061		-20.3038010574707			
1062		-20.4066388206539			
1063		-20.0550316112940			
1064		-20.1696222616982			
1065		-20.1627664108194			
1066		-20.0089994696787			
1067		-20.4017417843119			
1068		-20.6211290124362			
1069		-20.3655037153806			
1070		-20.0256493932417			
1071		-20.2264278832661			
1072		-20.2097779597031			
1073		-20.1049813819830			
1074		-20.5143736201793			
1075		-20.3419979409388			
1076		-20.5692204272103			
1077		-20.3743183807963			
1078		-20.1294665636933			
1079		-20.2185926251188			
1080	-20.1625303208247	-20.4232887442169			
1081		-20.0853932366148			

1082	-20.2214966273642	-20.1970456652138			
1083		-19.9257498518637			
1084	-19.7291279677599	-20.0276082077785			
1085		-19.8140974232647			
1086	-19.5679533965521	-20.2117367742400			
1087		-19.8944088192745			
1088	-20.0691670021373	-19.8689442302958			
1089		-20.4105564497276			
1090	-19.5139009488910	-20.2058603306295			
1091		-20.3792154171384			
1092	-20.0229767286814	-19.9306468882057			
1093		-20.0746197566623			
1094	-19.4912971980509	-20.1500341163300			
1095					
1096	-19.7468178597217	-20.3909683043594			
1097		-20.6838110776145			
1098	-19.6387129643994	-20.4908678457374			
1099		-20.3704007517227			
1100	-20.2716179879227	-20.1108578255935			
1101		-20.3390597191335			
1102	-19.7871115025237	-20.1226107128145			
1103		-20.0481757604152	-19.0921900377626		
1104	-19.8608193856980	-19.8699236375642			
1105		-20.0364228731942	-18.7149964530923		
1106	-19.7163519346764	-20.1627664108194			
1107		-20.3008628356654	-18.2031642864279		
1108	-19.7733526976645	-20.6975227793723			
1109		-20.5848909435049	-18.5659690150073		
1110	-20.1507370595168	-20.5613851690630			
1111		-20.3596272717702	-19.2802729423802		
1112	-19.9296134099940	-20.3145745374232			
1113		-20.3184921664969	-18.6779965374298		
1114	-19.9355100406479	-20.4722591076375			
1115		-20.2303455123398	-18.7417186144041		
1116	-19.6564028563613	-20.1970456652138			
1117		-20.3214303883021	-19.5577723098489		
1118	-20.1340299393307	-20.5584469472578			
1119		-20.2793158757604	-18.7170520039624		
1120	-19.9895624883091	-20.0707021275886			
1121		-19.7984269069701	-19.4066893208937		
1122	-19.7025812777317	-20.1118372328619			

1123		-19.8454384558539	-19.1744120725681		
1124	-19.1303498279665	-20.1392606363775			
1125		-20.3253480173757	-19.3522172228351		
1126	-19.1611149596743	-20.1950868506770			
1127		-20.0775579784675	-19.4611614189524		
1128	-19.0739470865022	-20.0697227203202			
1129		-20.1490547090616	-19.0993844658081		
1130	-19.0575390162581	-20.4321034096327			
1131		-20.1627664108194	-19.1805787251785		
1132	-19.6390000055356	-20.1186930837408			
1133		-19.8904911902008	-18.8373017298655		
1134	-19.7159128348051	-19.6456393730978			
1135		-19.5986278242140	-18.7006075970013		
1136	-19.8184632738311	-19.2959909782747			
1137		-19.4282109595104	-19.5859893954329		
1138	-19.4913273733381	-19.5486780535250			
1139		-19.4585725848311	-18.3059418299348		
1140	-18.8155199801567	-19.5506368680619			
1141		-19.4938312464940	-18.1291644551030		
1142	-18.9672946299152	-19.9198734082532			
1143		-19.8170356450699	-18.3692501531816		
1144	-19.3733943684582	-19.9698231789422			
1145		-20.2959657993234	-18.0709826649894		
1146	-19.0021617791840	-19.8806971175167			
1147		-19.9512144408424	-19.1951425428999		
1148	-19.4615877460206	-20.1539517454036			
1149		-19.8092003869226	-18.4803290798186		
1150	-19.3795473947998	-19.8826559320535			
1151		-19.7612094307704	-18.9246447863670		
1152	-19.2882775040666	-20.1970456652138			
1153		-20.3821536389436	-18.6150637037951		
1154	-19.3087875918718	-20.4605062204166			
1155		-20.5981406333079	-19.1632587769207		
1156	-19.8123102474895	-20.2802938113006			
1157		-20.7993811352871	-19.2218837659792		
1158	-19.9517788445649	-20.4397062174150			
1159		-20.0309063048025	-18.7611119221513		
1160	-19.8143612562701	-20.5910574214527			
1161		-20.2544041710260	-19.2157127144993		
1162	-19.6246429440719	-20.2231783622908			
1163		-20.1421864208838	-19.3298771668763		

1164	-19.6502805538284	-20.1597509382974			
1165		-19.8835976922953	-18.8557347115088		
1166	-19.6318214748037	-20.0426541555403			
1167		-20.2797751406234	-18.9688706553058		
1168	-19.7343719138298	-20.2456219123193			
1169		-19.9587347945644	-19.0604079189234		
1170	-19.1631659684549	-20.3236864341573			
1171		-20.0972993208269	-18.9873838097453		
1172	-19.4349246318738	-20.1890251339867			
1173		-20.5930090344986	-19.1519451825410		
1174	-19.5949033167544				
1175		-20.6027670997284	-19.0861206334227		
1176	-19.2913540172374	-19.9177509205994			
1177		-20.1168154512865	-18.8968750547077		
1178	-19.0575390162581	-20.0602186729539			
1179		-19.9284847923521	-18.2777128895640		
1180	-19.3149406182134	-19.8592025292209			
1181		-19.9743476989320	-18.5975790579356		
1182	-20.0979537485051	-19.9470251162887			
1183		-20.1051057730108	-18.6703949698585		
1184	-19.8704353542749	-19.8728638205425			
1185		-19.6406218680743	-18.7975621333782		
1186	-19.2749306876378				
1187		-19.8045573639342	-18.7497472798948		
1188	-19.8694461438652	-19.8211460748248			
1189		-19.7186863899124	-18.9380153979066		
1190	-19.3352725226293	-20.1119364186716			
1191		-19.7099041312056	-19.1025767707023		
1192	-19.7527193155211	-20.0036218946213			
1193		-19.8201702683018	-19.0758355476230		
1194	-19.2333838504306				
1195		-20.3314928863411	-18.6562040469940		
1196	-19.8427374628034	-19.9040896292777			
1197			-18.7518553449315		
1198	-19.4361719844183	-20.1860977144177			
1199		-19.8640815618357	-18.6490044869342		
1200	-19.9594642911476	-19.9928880228685			
1201		-19.9470251162887	-18.8691053230484		
1202	-19.5242117108813	-19.8309041400546			
1203		-19.8875009183872	-18.8279649798495		
1204	-19.8882411416495	-19.6601379985338			

1205		-19.7908960726126	-18.8979035632877		
1206	-19.6597335370096	-19.9382428575819			
1207			-18.9740131982057		
1208	-19.2215133255142	-20.0221622185578			
1209		-20.1656057774352	-18.6205454417588		
1210	-19.5914780187406	-20.2505009449341			
1211		-20.0475331881552	-18.5045689886289		
1212	-19.8575756189489	-20.1226702904243			
1213			-19.3483903213158		
1214	-19.8387806211647	-19.7821138139058			
1215		-19.9762993119780	-19.1406315881613		
1216	-19.8239424650192	-20.2182993296759			
1217		-19.9314122119211	-18.8485351514490		
1218	-19.9100037706628	-19.7538154247395			
1219		-20.1226702904243	-18.5656952919564		
1220	-19.7804172069926	-19.8972589836169			
1221			-19.0758355476230		
1222	-19.6894098493005	-20.0865654490742			
1223			-19.1159473822419		
1224	-19.7002911638072	-20.2719686884396			
1225			-19.0501228331237		
1226	-20.0633313841657	-20.4378557973455			
1227		-20.2007348122624	-19.0820065991028		
1228	-19.7744819445344	-20.2368396536125			
1229		-20.4651783799888	-19.2486249890585		
1230	-19.9980434971258	-20.0553396403390			
1231		-20.0680251251377	-19.3103355038568		
1232	-20.0415687551524	-20.2631864297328			
1233		-20.1529202926366	-18.8145943683099		
1234	-19.9545182390991	-20.2036622318313			
1235			-18.8026488199190		
1236	-19.9673779744252	-20.1636541643893			
1237		-19.8826218857723	-19.0994912449624		
1238	-19.9812269201609	-20.0553396403390			
1239		-19.9519041489036	-19.0840636162628		
1240	-19.8892303520592	-19.8757912401115			
1241		-19.7869928465207	-18.4978137256782		
1242	-19.9600332544687	-19.6191541245688			
1243			-18.5389540688771		
1244	-19.7972590279046	-19.5781702506039			
1245		-19.5567025070984	-18.7251141218522		

1246	-20.1297130542810	-19.3644686220722			
1247		-19.6640412246257	-19.1653157940806		
1248	-19.4273176038953				
1249		-20.2524525579801	-18.6592895727339		
1250	-19.4263310934313	-19.9870331837307			
1251		-19.9197025336454	-18.0864102936890		
1252	-19.3523428086294	-19.9694686663171			
1253			-18.7878531452306		
1254	-19.9008426266272	-19.8894525314331			
1255		-19.9118960814616	-19.0922916849026		
1256	-20.1731195146981	-19.6855089681312			
1257		-19.7869928465207	-18.8217939283697		
1258	-20.1376051379932	-19.8484686574681			
1259			-18.5718663434362		
1260	-19.7173516803186	-19.8806702727263			
1261		-19.5967105745404	-18.5122128457978		
1262	-19.8505305929620	-19.6933154203150			
1263		-19.7167347768664	-18.3651361188617		
1264	-19.8860449696669	-19.9480009228117			
1265		-20.0338718968335	-18.5327830173973		
1266	-20.1454972217054	-19.8787186596804			
1267		-20.0875412555972	-18.4361032108798		
1268	-20.0172508613822	-19.8914041444791			
1269		-19.6913638072690	-18.8588202372487		
1270	-19.6275792280923	-19.9372670510589			
1271			-18.2849124496238		
1272	-19.4776296375606	-20.0777831903674			
1273			-18.0689256478294		
1274	-19.5683886002508	-19.8855493053412			
1275			-18.7199715789523		
1276	-19.4164659887910	-20.2085412644462			
1277		-20.0524122207701	-18.9092171576674		
1278	-20.0241564346303				
1279		-19.6347670289364	-18.8952265149614		
1280	-19.9580602335407	-19.4435089504332			
1281		-19.6884363877001	-18.8063662996701		
1282	-18.9814148741560				
1283		-19.6708718702866	-18.4903262663147		
1284	-19.4539533864240	-19.7918718791355			
1285		-19.5771944440809	-17.7274607992784		
1286	-19.5535909432905	-19.7665009095382			

1287		-19.5567025070984	-18.4258181250801		-20.3831801793107
1288	-19.7143921489265				-20.6271575271669
1289		-19.8035815574112	-17.5793555637623		-20.6054476784170
1290	-18.6677045465961	-19.7688049254242			-19.9065973091340
1291		-19.8221218813478	-17.9959015386514		-19.3473102532434
1292	-19.6936754291820	-19.6894121942231			-20.1960619591329
1293		-19.6669686441946	-17.7799147368570		-20.0978507385976
1294	-19.5999569350996	-19.8353082604903			-20.6881518641309
1295		-19.6712746759903	-18.1882326431063		-20.6695434223453
1296	-19.7528660570235	-19.5886669847632			-20.4689857719889
1297		-19.6339045299590	-17.8416252516554		-19.7349861237776
1298	-20.0715089369035	-19.7322470195151			-20.3583689235965
1299		-19.6034183581966	-18.8166513854698		-20.4958646323460
1300	-19.3730595283739	-19.6594735772436			-20.5806364227028
1301		-19.5719487615386	-18.2057172889659		-19.9696592507409
1302	-19.8386924673937	-19.6142360320477			-20.8204785612732
1303			-18.2828554324638		-20.4989660393102
1304	-19.6236331862362	-19.4254184521000			-20.2829013541325
1305			-18.3332523528825		-20.1784873196687
1306	-19.4381692189996	-19.6555398776613			-20.1691830987758
1307			-18.4998707428381		-20.1619464825259
1308	-19.5299146921539	-19.6073520577788			-20.1236957966332
1309		-19.4116505035622	-18.2365725463650		-20.0585662503834
1310	-19.3809516120861	-19.5257277914473			-19.6946678332420
1311		-19.3998494048154	-18.5975790579356		-20.0813099014548
1312	-19.6917024082540	-19.1992307261210			-20.2281098310970
1313		-19.3772306322175	-18.5482106460969	-20.7417569581361	-20.3842139816321
1314	-19.5950243827795			-20.2717475612530	
1315		-19.1895862629241	-18.0843532765290	-20.6100732909059	
1316	-19.3454372353812	-19.1257060975064		-20.4834543801076	-20.3335576678823
1317		-19.2770529509576	-18.1018379223886	-19.7399481359004	-20.2053661800257
1318	-19.3148554109965			-20.3649390796005	-19.9076311114555
1319		-19.3409331163753	-18.4402172451997	-20.7387181042769	-20.0409916109192
1320	-19.4944003154490			-20.1917244096285	-19.8631776116342
1321		-19.6308507901942	-18.5482106460969	-20.7478346658544	-20.1567774709187
1322	-19.4805891689526			-20.1400638940228	-20.0926817269904
1323		-19.7124208475737	-18.4957567085182	-20.8977514562395	-19.9696592507409
1324	-19.7775288186241			-20.9251011409719	-19.9407127857410
1325		-19.7664732952349	-18.0318993389504	-20.4449622312250	-19.9045297044912
1326	-19.7035405338223			-20.5270112854222	-19.9045297044912
1327		-20.1045467860610	-18.4597589082192	-20.5989308267556	-19.7060396587777

1328	-19.4894677631289	-19.8598366139223		-19.7085466460224	-19.9975719134194
1329		-19.9423894430775	-18.0596690706097	-20.2454108278069	-19.8280283327058
1330	-19.8386924673937	-19.7369901419652		-21.0740049800706	-20.2839351564540
1331		-19.2839323533872	-18.1450352827474	-21.2786211399206	-20.0875127153833
1332	-20.0350080497346			-21.2067015985872	-19.7215466935991
1333		-19.2033450677833	-18.6808882529134	-21.0517200517702	-20.0699380759191
1334	-20.0073857567419			-20.7508735197135	-20.2549886914541
1335		-19.3448642034779	-18.3342808614625	-20.6982000528215	
1336	-19.8603956976022	-19.5482979610390		-20.8430520867746	-20.2177718078828
1337		-19.5217631230962	-18.5471821375169	-21.0466552953382	-20.1877915405615
1338	-19.4105469260069			-20.4702860133846	-19.9593212275267
1339		-19.7242141088816	-18.3733641875015	-20.7346662991314	-19.6957016355634
1340	-19.2596108250111			-20.1258825760134	-19.4258792296717
1341			-18.4011339191607	-20.4966227468307	-19.4308952925654
1342	-19.3069633272843			-20.2930195382671	-19.3451441448188
1343			-18.5656952919564	-20.5938660703237	-19.4091992190392
1344				-19.8392173619662	
1345			-18.4134760221204	-20.4328068157883	-19.7325740292164
1346				-20.3770944950371	
1347			-18.6099211608952	-20.0802997681260	-19.8462201286397
1348	-19.6457436662665			-20.3527836641639	-20.4196163575481
1349			-18.4546163653193	-20.1907114583421	-20.4392461383576
1350	-19.9678621753372			-20.6776014788533	-20.7078641915400
1351			-18.3291383185626	-20.3459495163940	-20.1313685235563
1352	-19.6467566175529			-20.4019030683426	-20.2160865249446
1353			-18.3805637475613	-20.3011866748351	-19.9598662280630
1354	-19.3854151856653			-20.0855111655057	
1355			-18.2941690268435	-20.6338559746026	
1356	-19.3459100854962			-19.8667836442518	
1357			-18.3116536727031	-19.8708529934844	
1358	-19.7217150127454			-20.5494169780255	-19.7170768338405
1359			-17.6246099412811	-19.7609805642034	-19.6581874914120
1360	-19.4117519191113			-20.0865285028138	-19.5393756601968
1361			-18.1326931797878	-20.4578566202913	-19.6271931006602
1362	-19.0187268199936			-19.7731886119013	-19.7625352736098
1363			-18.1094045896575	-20.4059724175753	
1364	-19.6396659585482			-19.9298585573576	
1365			-18.7902595260970	-21.2544317325787	-19.9288718373112
1366	-19.1362291692143			-20.1740195113154	
1367			-18.4348965108600	-20.7355897054183	-19.7762406238258
1368	-19.5748370762195			-20.5392436049439	-19.5476408310639

1369			-18.3803045114178	-20.7325376934938	
1370	-19.4066871626794			-20.0916151893546	-19.8493195677149
1371			-18.0980741746789	-20.5066888110829	-19.9381701545367
1372	-18.9042633246320			-20.2493024721190	-19.5796683681741
1373			-18.2412494184990	-20.6633587565391	-19.9340375691031
1374	-19.4411164426155			-20.4181804652732	-19.7790656153441
1375			-17.7880328193562	-20.5056714737747	-19.5755357827405
1376	-19.0453569290535			-19.8474542353968	-19.7924965180032
1377			-18.1238251178120	-20.3194987463819	-19.8482864213565
1378	-19.8067419830636			-20.6796361534697	-20.3070034044832
1379			-18.4194459449801	-20.3215334209982	-20.6190136047181
1380	-19.9353136016828			-20.6084225418986	-20.6293450683020
1381			-17.9013369691419	-19.9349452438983	-20.4909034562773
1382	-19.5546210746777			-19.2767280055204	
1383			-17.9415084404296	-19.2380691878104	-20.4382129919992
1384	-19.3838618936992			-20.4853247276116	-19.7821650544193
1385			-18.5286299438645	-20.7732311858201	-19.7181099801989
1386	-19.4742638130407			-20.5595903511071	-19.9577999353462
1387			-18.2350691921471	-19.7324951195750	-19.5466076847055
1388	-19.3527234548148			-20.4364925368200	-20.2367494521125
1389			-18.4585873785425	-20.0905978520464	-20.1127718891052
1390	-19.1618749584271			-19.8525409219376	-20.1324016699147
1391			-18.5172995288860	-20.2564238332761	-20.1107055963884
1392	-19.7193534610334			-20.4171631279650	-20.6200467510765
1393			-18.4709478312464	-20.0183669031673	-20.2109207931526
1394	-19.3416743313398			-20.3957990444937	-20.7057978988232
1395			-18.9210743172132	-20.3072906986840	-20.8762670479581
1396	-19.0835266283310			-20.4202151398895	-20.8587035598654
1397			-18.2639102484561	-20.3398454925450	-20.6107484338510
1398	-19.3145537555373			-20.0926325266628	-20.4516438946583
1399			-18.4060554545509	-20.3520535402429	-20.5539253841393
1400	-19.3617636467490			-20.5524689899500	-20.5270635788211
1401			-18.2670003616321	-19.8240554773092	-20.2822078918818
1402	-19.7173445294925			-20.3205160836900	-20.3793236495708
1403			-18.4699177935210	-20.2482851348109	-20.6923669961641
1404	-19.5636612666118			-20.6552200580739	-20.7057978988232
1405				-20.3103427106085	-20.7006321670312
1406	-19.5626568008414			-20.8454621346993	-20.6107484338510
1407			-19.2259654839092	-20.9512652147477	-20.2284842812453
1408	-19.3918976198629			-20.2849092779045	-20.1933573050599
1409			-18.8551519027924	-19.8525409219376	-20.2388157448293

<table>
<tr><td>1410</td><td>-19.4772772103521</td><td></td><td></td><td>-20.3133947225329</td><td>-20.5652899940816</td></tr>
<tr><td>1411</td><td></td><td></td><td>-18.1464859477692</td><td>-18.3906272101152</td><td rowspan="2">-19.8668830558075</td></tr>
<tr><td>1412</td><td>-18.7570752529309</td><td></td><td></td><td>-19.2746933309041</td></tr>
<tr><td>1413</td><td></td><td></td><td>-18.4688877557957</td><td>-20.9858546832251</td><td>-19.7026127848230</td></tr>
<tr><td>1414</td><td>-19.3667859756013</td><td></td><td></td><td>-21.0428255724819</td><td>-19.9701976916469</td></tr>
<tr><td>1415</td><td></td><td></td><td>-18.8788427704749</td><td>-22.0092960152315</td><td>-19.7650499134360</td></tr>
<tr><td>1416</td><td>-19.2201339731139</td><td></td><td></td><td>-21.1465939779140</td><td>-20.2346831593957</td></tr>
<tr><td>1417</td><td></td><td></td><td>-18.9056237513333</td><td>-20.4731166799137</td><td>-20.0363190585841</td></tr>
<tr><td>1418</td><td>-19.0805132310196</td><td></td><td></td><td>-20.3774869729469</td><td>-19.8792808121083</td></tr>
<tr><td>1419</td><td></td><td></td><td>-18.8623621668697</td><td>-20.8505488212401</td><td>-20.0270207413586</td></tr>
<tr><td>1420</td><td>-19.1679017530498</td><td></td><td></td><td>-20.4171631279650</td><td>-19.8947780074842</td></tr>
<tr><td>1421</td><td></td><td></td><td>-18.4328364354094</td><td>-19.7141830480282</td><td>-19.9113083492185</td></tr>
<tr><td>1422</td><td>-19.1699106845908</td><td></td><td></td><td>-19.1343007823784</td><td>-19.6492443235700</td></tr>
<tr><td>1423</td><td></td><td></td><td>-18.3607337946366</td><td>-19.5697211502698</td><td>-19.9842991154025</td></tr>
<tr><td>1424</td><td>-19.3878797567810</td><td></td><td></td><td>-20.0814418162730</td><td>-19.7201608285897</td></tr>
<tr><td>1425</td><td></td><td></td><td>-18.3741242850659</td><td>-19.4690047567622</td><td>-19.5916888992061</td></tr>
<tr><td>1426</td><td>-19.5455808827435</td><td></td><td></td><td>-20.0804244789649</td><td></td></tr>
<tr><td>1427</td><td></td><td></td><td>-18.5275999061392</td><td>-19.8433848861642</td><td>-19.5022724363551</td></tr>
<tr><td>1428</td><td>-19.3647770440604</td><td></td><td></td><td>-19.3743923871036</td><td rowspan="3">-19.7139941759793</td></tr>
<tr><td>1429</td><td></td><td></td><td>-18.7789291111184</td><td>-19.7640325761279</td></tr>
<tr><td>1430</td><td>-19.4853129365158</td><td></td><td></td><td>-19.2848667039857</td></tr>
<tr><td>1431</td><td></td><td></td><td>-18.5574710001736</td><td>-20.1831755470888</td><td>-19.7458552144665</td></tr>
<tr><td>1432</td><td>-19.5164513754001</td><td></td><td></td><td>-20.2014876186356</td><td>-19.9061881823372</td></tr>
<tr><td>1433</td><td></td><td></td><td>-18.2711205125334</td><td>-19.9420666050554</td><td>-19.5937444500763</td></tr>
<tr><td>1434</td><td>-19.2804019193416</td><td></td><td></td><td>-19.8759396800252</td><td>-19.9914935434480</td></tr>
<tr><td>1435</td><td></td><td></td><td>-19.0549792215054</td><td>-20.4619259695239</td><td>-20.4128814718262</td></tr>
<tr><td>1436</td><td>-19.4491521687792</td><td></td><td></td><td>-20.3062733613758</td><td>-20.0901599852146</td></tr>
<tr><td>1437</td><td></td><td></td><td>-18.7665686584145</td><td>-20.6308039626781</td><td>-20.2926317459232</td></tr>
<tr><td>1438</td><td>-19.3738172359945</td><td></td><td></td><td>-19.3693057005628</td><td>-19.8291050247071</td></tr>
<tr><td>1439</td><td></td><td></td><td>-18.4379866240360</td><td>-20.4011793658504</td><td>-19.8404105544928</td></tr>
<tr><td>1440</td><td>-19.4883263338272</td><td></td><td></td><td>-20.5640185123238</td><td>-19.7067997479339</td></tr>
<tr><td>1441</td><td></td><td></td><td>-18.4503470767399</td><td>-20.4011793658504</td><td>-19.9986879714934</td></tr>
<tr><td>1442</td><td>-19.2894421112757</td><td></td><td></td><td>-20.1887804791459</td><td>-20.2967428476634</td></tr>
<tr><td>1443</td><td></td><td></td><td>-18.5111193025340</td><td>-20.1240493898646</td><td>-20.1929375287215</td></tr>
<tr><td>1444</td><td>-19.4702459499589</td><td></td><td></td><td>-20.1098894640843</td><td>-20.2710484617867</td></tr>
<tr><td>1445</td><td></td><td></td><td>-18.5760116792295</td><td>-20.2535115684273</td><td>-20.2741317880919</td></tr>
<tr><td>1446</td><td>-19.7143311321811</td><td></td><td></td><td>-20.4092707520106</td><td rowspan="2">-20.0706322519483</td></tr>
<tr><td>1447</td><td></td><td></td><td>-18.5894021696587</td><td>-19.8499536836888</td></tr>
<tr><td>1448</td><td>-19.4350896479927</td><td></td><td></td><td>-19.9733473226314</td><td>-19.6225221622582</td></tr>
<tr><td>1449</td><td></td><td></td><td>-18.2494897203016</td><td>-19.9055819635400</td><td>-19.6564387516155</td></tr>
<tr><td>1450</td><td>-19.3828574279287</td><td></td><td></td><td>-20.0643754169333</td><td>-19.6214943868231</td></tr>
</table>

1451			-18.7469979416333	-20.4557962224315	-19.8979659788567
1452	-19.7394427764427			-20.4487162595414	-19.7890217827394
1453			-18.1845973436062	-20.6884235745364	
1454	-19.4119869352721			-20.3061055784684	-19.4909669065694
1455			-18.2154984753659	-20.0825810357937	
1456	-19.8549563400458			-20.6085211362047	-19.8671327158046
1457			-18.3679440587139	-20.7814745153784	
1458	-19.5747103900869			-20.2039518281963	-19.9072159577723
1459			-18.4925786234782	-19.7528570497668	
1460	-19.6831926932968			-20.1655177439354	-20.0696044765132
1461			-18.7542082057106	-20.8836282656505	-19.8578827368890
1462	-19.3908931540924			-20.4426477199213	-19.5444112291930
1463			-18.8561819405177	-20.4820932274521	-19.3624949771857
1464	-19.7454695710655			-20.5174930419028	-19.7376330109859
1465			-18.5997025469119	-20.6156010990949	-19.9164659366879
1466	-19.5706925270051			-20.4638876085917	-19.9709380347466
1467			-18.7696587715905	-20.4244421010609	-20.2062986093774
1468	-19.9885502875173			-20.7157320028270	-20.2361040969944
1469			-18.4019353036496	-20.0593183005832	-20.0038268486688
1470	-19.7213623925744			-20.1908033256860	
1471			-18.3638239078126	-20.3860080168001	
1472	-19.7263847214267			-20.5356986607632	-19.8599382877591
1473			-18.0342118357088	-19.8529879534989	
1474	-19.8840858473892			-20.4305106406810	
1475			-18.5028790007314	-20.4426477199213	-19.9329103436490
1476	-19.3085269609145			-20.0158270999723	-19.8003273125251
1477			-18.1598764381984	-19.6972287699156	-19.9668269330063
1478	-19.4812950734340			-19.8469194138788	-19.7725773757783
1479			-18.7614184697879	-20.2322716797568	-19.9956046451882
1480	-19.7514963656882			-19.5960864429135	-20.0192434801948
1481			-18.2288889657951	-20.2231688703267	-19.7304385829404
1482	-19.1538392322634			-20.2262031401367	
1483			-18.5255398306886	-20.5235615815229	-19.5927166746412
1484	-19.1829687396068			-20.3050941551984	-19.7160497268495
1485			-18.3133520592717	-19.9672787830113	-19.5341334748423
1486	-19.8529474085049			-20.5933497871544	-19.8815215718956
1487			-18.3833946245938	-19.7831997478674	-19.6944664427130
1488	-19.0855355598720			-18.4481210314394	
1489			-17.6211667078536	-18.3459672811673	-19.4488281137315
1490	-18.8585262957476			-18.4177783333388	-18.9061626840151
1491			-16.9660627145472	-18.6069144848328	-18.6451077235076

1492	-19.2271652335071			-19.1793800556648	-19.0459401431845
1493			-17.4625408981537	-18.9932781739809	-19.1004122412432
1494	-18.7982583495198			-19.1227403525436	-18.9883847188206
1495			-17.8477750074250	-19.3108650807675	-19.2124397636657
1496	-19.1297320537723			-19.8408508742586	-19.3614672017507
1497			-18.2494897203016	-19.8216338321282	-19.3624949771857
1498	-19.4270539218290			-19.3867218260191	-19.6502720990051
1499			-17.4419401436472	-19.9612102433912	
1500	-19.3738172359945			-19.6517147227647	-19.5166612924461
1501			-18.0403920620607	-19.5111268882317	-19.6841886883623
1502	-19.4732593472703			-19.6800345743253	-19.4786336013485
1503			-18.2886311538639	-19.3088422342275	-19.6821331374922
1504	-19.4069646064198			-19.2309626424359	
1505			-18.1444258723185	-19.3513220115684	-19.5701056150697
1506	-19.5676791296937			-19.2623167638065	-19.5074113135305
1507			-18.1413357591425	-19.4767384970510	-19.1065788938536
1508	-19.6631033778876			-19.5910293265634	-19.1384399323407
1509			-18.0383319866101	-19.8802963817895	-19.3439950193545
1510	-19.6751569671331			-20.0744896496335	-19.3337172650038
1511			-18.2618501730055	-20.0997752313841	-19.2864395949906
1512	-19.5204692384820			-19.8610793396591	
1513			-18.9406450339944	-20.2747514570977	-19.4488281137315
1514	-19.9282823412895			-20.1442778552650	-19.4991891100499
1515			-18.3947250395724	-20.1149465804344	-19.5999111026867
1516	-19.4551789634020			-20.0299870257526	-20.0408267643313
1517			-18.5842519810321	-20.6813436116463	-19.8527438597136
1518	-19.8268312984729			-20.3597110117795	-19.1846898269188
1519			-18.0383319866101		-19.3840782613222
1520	-19.7866526676544				
1521			-18.3566136437354	-20.0633639936633	-19.4961057837447
1522	-19.6631033778876			-20.6236924852550	-19.7376330109859
1523			-17.9713795344640	-20.0967409615740	-20.1045488413055
1524	-19.6681257067399				-20.2206874654683
1525			-18.0640829297432		-19.8753549192852
1526	-19.6460274597897			-20.3880308633401	
1527			-18.3895748509457		
1528	-19.0885489571833				-19.9709380347466
1529			-18.2062281358380	-20.3131855413585	
1530	-19.7143311321811			-20.3597110117795	-20.1847153252409
1531			-18.1763570418036	-20.4770361111020	
1532	-19.5144424438592			-20.5296301211431	-20.1867708761111

1533			-18.3700041341646	-20.5822241311842	-20.5166867907682
1534	-19.3627681125195			-20.1493535442253	
1535			-18.1495760609451	-20.2492231560847	-20.2802984407023
1536	-19.5234826357934			-20.8787043459865	-20.3203816826700
1537			-18.1825372681555	-20.3299258727388	-20.0418545397663
1538	-19.2131027127206			-19.9909744627917	-20.0839933326042
1539			-18.3627938700873	-20.1079934019401	-19.8938548771164
1540	-19.5064067176955				-19.8599382877591
1541			-17.9033970445926	-20.4308042685564	-19.7016608707585
1542	-19.4511611003201			-20.4802346825070	
1543			-18.1667220262287	-20.1372481367272	-20.0120490521493
1544	-19.8840858473892			-21.1974800767701	
1545			-18.5996082395620	-21.1884010211465	
1546	-19.8238179011615			-21.2529631944697	
1547			-18.2175302672303	-20.4640941391762	
1548	-19.5666746639232			-20.3218556010734	
1549			-18.3719873198750	-20.3642245273168	
1550	-19.5706925270051			-20.9553719268079	
1551			-18.0427499181849	-20.6769475543513	
1552	-19.7468216858963			-20.3773387187731	
1553			-18.3445508697342	-20.3077326256589	
1554	-19.3140456805905			-20.7626941907963	
1555			-18.1352209168078	-20.2088717977577	
1556	-19.8826932224458			-20.5589198312447	
1557			-18.5467676689203	-20.1614589517234	
1558	-19.9058417064505			-21.0945841130361	
1559			-18.1047359722068	-20.4509799477199	
1560	-19.5918274886473			-20.8050631170396	
1561			-18.4126339126762	-20.4741819787579	
1562	-19.7126021878023			-20.0858001548602	
1563			-18.1220107741474		
1564	-19.5747177396003			-20.1856697667196	
1565			-18.3221952436935	-20.9301523278535	
1566	-19.5163433016753			-21.0199341001311	
1567			-18.3293083974337	-20.4560238675108	
1568	-19.0815543847170			-19.8517622765634	
1569			-18.2480152118312	-20.6981320174730	
1570	-19.4156977190461			-20.8040543330815	
1571			-18.3191467492334	-19.7902264551147	
1572	-19.2979423873699			-20.8696252903629	
1573			-18.3282922326137	-20.5326914483321	

1574	-19.6371180008304			-20.8837482657774	
1575			-18.1931423115495	-20.9876530134695	
1576	-19.3231037830272			-20.7172989126783	
1577			-18.2744354971520	-21.0330482915874	
1578	-19.7186409227601			-21.1752868296902	
1579			-18.2378535636309	-20.4055846696020	
1580	-19.9118804414082			-20.9543631428497	
1581			-18.4197470664165	-20.8080894689142	
1582	-19.7055569970183			-21.1319091194886	
1583			-18.6138345470424	-21.0431361311692	
1584	-19.4177106306987			-20.8928273214009	
1585			-18.3831651328953	-20.8312914999522	
1586	-19.9823323492487			-21.0522151867927	
1587			-18.6260285248828	-20.8726516422374	
1588	-19.5797500187317			-21.0955928969943	
1589			-18.7754047534273	-20.6658509308114	
1590	-19.3341747971164			-20.0777298831948	
1591			-18.6107860525823	-20.5811130783246	
1592	-19.6008855910839			-21.1591462863594	
1593			-18.4400703628171	-20.8363354197431	
1594	-19.5213755808068			-20.4994015777123	
1595			-18.6672327236177	-20.6466840356060	
1596	-19.6381244566567			-20.5256299606249	
1597			-18.1627829829975	-20.6345786281079	
1598	-19.0865866638485			-20.5538759114538	
1599			-18.3815804608568	-20.5659813189520	
1600	-19.7126021878023			-20.7243604003856	
1601			-18.3856322660024	-20.5569022633284	
1602	-19.6743568664032			-20.4842698183397	
1603			-18.7269968495144	-20.1019406981910	
1604	-19.3492716345108			-20.4741819787579	
1605			-18.5406138128194	-20.5659813189520	
1606	-19.5616338138585			-20.6265083564425	
1607			-18.0462935850631	-20.5337002322903	
1608	-20.1725525004180			-21.1329179034468	
1609			-18.3339717503967	-20.4358481883473	
1610	-19.3231037830272			-19.3070189391484	
1611			-18.1030188571007	-20.1281690811036	
1612	-19.4821238035814			-20.5034367135450	
1613			-18.2549615500586	-20.7283955362183	
1614	-19.1359029993368			-20.4368569723054	

1615			-18.1607570804247	-20.8312914999522	
1616	-19.4680334220133			-19.8043494305291	
1617			-18.5375749589603	-20.7526063512145	
1618	-19.2144065537876			-20.5609373991611	
1619			-18.1931715215890	-20.1453184083926	
1620	-20.2983594787045			-19.8921136348904	
1621			-18.4109560481620	-20.5790955104082	
1622	-19.6391309124830			-19.8810170113505	
1623			-18.2083657908848	-20.4600590033435	
1624	-19.4177106306987			-20.4237427808491	
1625			-18.3481530684061	-20.2431704523357	
1626	-18.9939927278295			-20.2966360021190	
1627			-18.6672327236177	-19.8447007888562	
1628	-19.5274143157645			-19.3332473220610	
1629			-18.5092123229414		
1630	-19.2214517445716			-20.7334394560092	
1631			-18.3015573092323	-20.5044454975032	
1632	-19.1660966741256			-21.0246551331591	
1633			-18.6094945002937	-20.7144587262309	
1634	-18.8108177674443			-20.0546598541654	
1635			-18.2782594296455	-19.7080886242750	
1636	-19.0241864026183			-20.1263990883410	
1637			-18.1496146162744	-19.9313896207933	
1638	-18.7745853576978			-19.6312973313547	
1639			-18.3846193147160	-20.3426272026167	
1640	-18.9778894346089			-20.4871160827168	
1641			-18.1070706622462	-20.1951070872697	
1642	-18.9627925972145			-19.9505874440234	
1643			-18.5051605177959	-19.5181312154721	
1644	-18.9285730991205			-19.6181619786183	
1645			-18.6408959901716	-20.1061908533620	
1646	-18.9577603180830			-20.4376059070182	
1647			-18.7138284827914	-20.9357388992514	
1648	-19.2586906101445			-20.5416783171602	
1649			-18.2245730114670	-20.1850029697802	
1650	-19.4479043054874			-19.9425041500318	
1651			-18.6338053311669	-19.9435145617808	
1652	-19.3070004898065			-20.7265836672184	
1653			-18.5436526666786	-20.7579064314359	
1654	-19.1902516139566			-20.4770119652273	
1655			-18.2812982835046	-20.4658974359888	

1656	-19.2697616242337			-20.2971586739139	
1657			-17.8953638433916	-20.3850644960727	
1658	-18.8681857495430			-20.0607223246592	
1659			-18.2255859627534	-20.0910346771277	
1660	-19.2385614936186			-20.3507104966083	
1661			-18.2863630399365	-20.5012618472021	
1662	-19.0926253988062			-20.4143664367923	
1663			-18.2853500886502	-20.5618865521392	
1664	-19.2969359315436			-20.2557317922068	
1665				-20.3769812020811	
1666	-19.2607035217970			-20.1971279107676	
1667				-21.0084885451759	
1668	-19.1711289532570			-20.4585886355796	
1669				-20.2221805596101	
1670	-18.9990250069610			-20.5909367464855	
1671			-18.2742076244999	-20.5303192911088	
1672	-19.9450934836759			-20.4757635812697	
1673			-18.5031346152231	-19.8998977551903	
1674	-19.2395679494449				
1675			-18.6145592567256	-20.5151649272646	
1676	-19.8766544874880			-20.5959882011003	
1677			-18.2306507191853	-20.7475318395422	
1678	-19.4770915244499			-19.9847621927178	
1679				-20.2433966689920	
1680	-19.2315163028346			-20.9303944965955	
1681			-18.1769643010069	-20.6343792561722	
1682	-18.8141106601436			-20.5485045277218	
1683				-20.1272132128532	
1684	-18.9194575939278			-20.0958941942418	
1685				-20.1868203773070	
1686					
1687				-19.9019183370362	
1688	-19.2101746131205			-19.8231156450463	
1689				-20.4626297992714	
1690	-19.3155215469047			-20.9354459512102	
1691				-20.3737241980521	
1692	-19.5647075633556			-20.2151085231495	
1693				-21.1566996633355	
1694	-19.9577326624733				
1695				-19.8645375728871	
1696	-19.7470387949051			-20.2757259785263	

1697				-19.4775961493987	
1698	-19.3236251571957			-19.2219925458933	
1699				-20.7354083484669	
1700				-20.0403281934798	
1701				-20.6727703112442	
1702	-19.1585140975148			-20.4757635812697	
1703				-20.4252490351223	
1704	-19.8665670466986			-20.4707121266549	
1705				-20.1080176853172	
1706	-19.7855309437877			-19.9241447373410	
1707				-19.7331997529041	
1708	-19.6335882508298			-20.2666333602198	
1709				-20.6818629295507	
1710	-19.6801840100036			-20.4131255440470	
1711				-19.7948274992038	
1712	-19.8199712875249			-19.8271568087381	
1713				-20.5272884183399	
1714	-20.3082138075629			-20.6242763469428	
1715				-19.7736113898220	
1716				-19.6735925884503	
1717				-20.4043991164885	
1718				-19.9122108889621	
1719				-19.3778350990764	
1720				-19.3989288802561	
1721				-19.5727014585460	
1722				-19.4451343056973	
1723				-19.8378804219480	
1724				-19.9875458217468	
1725				-19.1307365195427	
1726					
1727					
1728				-19.0011604351532	
1729				-18.5531687015271	
1730				-18.9519416124005	
1731				-19.7072998717879	
1732				-20.0116530002379	
1733				-19.9523895197806	
1734				-20.1964747020029	
1735				-20.0367646444994	
1736				-20.1783943181346	
1737				-20.2346444012804	

1738				-19.8740411896846	
1739				-20.4054035822590	
