# Supplementary material for: Severe multi-year drought coincident with Hittite collapse around 1198–1196 bc
Source: Nature. 2023 Feb 8;614(7949):719–24. doi: 10.1038/s41586-022-05693-y (PMC9946833; doi:10.1038/s41586-022-05693-y)
Supplement: Supplementary file 2 — Reporting Summary [file 41586_2022_5693_MOESM2_ESM.pdf]

nature portfolio

Corresponding author(s): Sturt W. Manning

Last updated by author(s): Nov 3, 2022

Reporting Summary

Nature Portfolio wishes to improve the reproducibility of the work that we publish. This form provides structure for consistency and transparency in reporting. For further information on Nature Portfolio policies, see our Editorial Policies and the Editorial Policy Checklist.

Statistics

For all statistical analyses, confirm that the following items are present in the figure legend, table legend, main text, or Methods section.

n/a	Confirmed	
☐	☒	The exact sample size (*n*) for each experimental group/condition, given as a discrete number and unit of measurement
☐	☒	A statement on whether measurements were taken from distinct samples or whether the same sample was measured repeatedly
☐	☒	The statistical test(s) used AND whether they are one- or two-sided<br>*Only common tests should be described solely by name; describe more complex techniques in the Methods section.*
☒	☐	A description of all covariates tested
☐	☒	A description of any assumptions or corrections, such as tests of normality and adjustment for multiple comparisons
☐	☒	A full description of the statistical parameters including central tendency (e.g. means) or other basic estimates (e.g. regression coefficient) AND variation (e.g. standard deviation) or associated estimates of uncertainty (e.g. confidence intervals)
☒	☐	For null hypothesis testing, the test statistic (e.g. *F*, *t*, *r*) with confidence intervals, effect sizes, degrees of freedom and *P* value noted<br>*Give P values as exact values whenever suitable.*
☒	☐	For Bayesian analysis, information on the choice of priors and Markov chain Monte Carlo settings
☐	☒	For hierarchical and complex designs, identification of the appropriate level for tests and full reporting of outcomes
☐	☒	Estimates of effect sizes (e.g. Cohen's *d*, Pearson's *r*), indicating how they were calculated

Our web collection on statistics for biologists contains articles on many of the points above.

Software and code

Policy information about availability of computer code

Data collection	No software was directly used for primary tree-ring sample data collection for this paper; tree-ring data previously assembled/measured and previously checked. This previous work used standard dendrochronological methods - references are given in the paper. This work used Tellervo and Corina software for tree ring measurement and data archiving (http://www.tellervo.org/, https://dendro.cornell.edu/corina/). The tree-ring width dataset obtained and used is provided in full in the paper. The stable carbon data used the equipment described and the associated standard Thermo Scientific Isodat Software Suite.
Data analysis	COFECHA 6.06P, ARSTAN (version 49v1b_MRWE) (both available from e.g. https://www.geog.cam.ac.uk/research/projects/dendrosoftware/ Software is described and referenced to the original publications in the paper. Other software used in the analysis: Microsoft Excel, OriginPro 2022b.

For manuscripts utilizing custom algorithms or software that are central to the research but not yet described in published literature, software must be made available to editors and reviewers. We strongly encourage code deposition in a community repository (e.g. GitHub). See the Nature Portfolio guidelines for submitting code & software for further information.

Data

Policy information about availability of data

All manuscripts must include a data availability statement. This statement should provide the following information, where applicable:

- Accession codes, unique identifiers, or web links for publicly available datasets
- A description of any restrictions on data availability
- For clinical datasets or third party data, please ensure that the statement adheres to our policy

All data are available in the Article or the Supplementary Information.

Human research participants

Policy information about studies involving human research participants and Sex and Gender in Research.

Reporting on sex and gender	N/A
Population characteristics	N/A
Recruitment	N/A
Ethics oversight	N/A

Note that full information on the approval of the study protocol must also be provided in the manuscript.

Field-specific reporting

Please select the one below that is the best fit for your research. If you are not sure, read the appropriate sections before making your selection.

☒ Life sciences ☐ Behavioural & social sciences ☐ Ecological, evolutionary & environmental sciences

For a reference copy of the document with all sections, see nature.com/documents/nr-reporting-summary-flat.pdf

Life sciences study design

All studies must disclose on these points even when the disclosure is negative.

Sample size	All available samples were employed. The Expressed Population Signal (EPS) measure (see Methods text for reference and level used) was employed to determine the period with adequate data for the ring-width analysis. Available population numbers by year are indicated in the relevant figures.
Data exclusions	Tree-ring width data. All data used. Analysis of ring-width data presented for the period with EPS at 0.85 or more. This level is widely used as an approximately satisfactory threshold in dendroclimate studies. Carbon isotope data. All data used except (as described in Methods) (i) the GOR-87 series was ended to exclude some data well after the time period of interest and (ii) the GOR-87 series is shown from RY1287 as data were corrupted during the times preceding these data.
Replication	All available data are used. Rare/unique archaeological material limits further replication. Two different but complementary methods are employed: tree-ring width and stable carbon isotope analysis. Both indicate similar results. This is our support for the general robustness of the findings reported.
Randomization	Not applicable. Data are time-series. They were analysed as such.
Blinding	Not applicable. Tree-ring samples collected at various times and measured (at various times) and chronology constructed using standard dendrochronological methods (by more than one worker and the original chronology was replicated by the different skeleton-plotting method in addition: see discussions in refs. 67 and 68). This cannot be a blind exercise as each sample and step relates to the other samples to construct a chronology. The validity of the crossdating is shown in Extended Data Fig. 2 based on COFECHA analysis (and the chronology is consistent with previous versions of the Gordion tree-ring chronology, see refs. 66-68). Analysis was then carried out on the time-series and on specific years using using (i) ARSTAN software and (ii) stable carbon isotope analysis (not blind--instead with very careful recording and procedures to ensure each specific sample was tracked and its place in the time-series kept secure).

Reporting for specific materials, systems and methods

We require information from authors about some types of materials, experimental systems and methods used in many studies. Here, indicate whether each material, system or method listed is relevant to your study. If you are not sure if a list item applies to your research, read the appropriate section before selecting a response.

Materials & experimental systems

n/a	Involved in the study
☒	☐ Antibodies
☒	☐ Eukaryotic cell lines
☒	☐ Palaeontology and archaeology
☒	☐ Animals and other organisms
☒	☐ Clinical data
☒	☐ Dual use research of concern

Methods

n/a	Involved in the study
☒	☐ ChIP-seq
☒	☐ Flow cytometry
☒	☐ MRI-based neuroimaging
